# Regulation of aerobic glycolysis to decelerate tumor proliferation by small molecule inhibitors targeting glucose transporters

**DOI:** 10.1007/s13238-020-00725-7

**Published:** 2020-05-15

**Authors:** Meng Gao, Jian Huang, Xin Jiang, Yafei Yuan, Huanhuan Pang, Shuchen Luo, Nan Wang, Chengbo Yao, Zuwan Lin, Debing Pu, Shuo Zhang, Pengcheng Sun, Zhuoyi Liu, Yu Xiao, Qian Wang, Zeping Hu, Hang Yin

**Affiliations:** 1 Key Laboratory of Bioorganic Phosphorus Chemistry and Chemical Biology (Ministry of Education), Department of Chemistry, Tsinghua University, Beijing, 100084 China; 2 Tsinghua University-Peking University Joint Center for Life Sciences, Tsinghua University, Beijing, 100084 China; 3 Beijing Advanced Innovation Center for Structural Biology, Tsinghua University, Beijing, 100084 China; 4 State Key Laboratory of Membrane Biology, School of Life Sciences, Tsinghua University, Beijing, 100084 China; 5 Department of Molecular Biology, Princeton University, Princeton, NJ 08544 USA; 6 School of Pharmaceutical Sciences, Tsinghua University, Beijing, 100084 China; 7 Laboratory Medicine Center, Zhujiang Hospital, Southern Medical University, Guangzhou, Guangdong 510515 China

**Dear Editor,**

Different from normal differentiated cells, metabolic reprogramming was spotted in cancer cells, due to increased demand for energy and macromolecule synthesis during their rapid proliferation (Hanahan and Weinberg, 2011; Pavlova and Thompson, 2016). Most cancer cells prefer anaerobic glycolysis even with oxygen in the environment due to its higher speed to produce macromolecular materials required for biosynthesis (Vander Heiden et al., 2009; DeBerardinis and Chandel, 2016). But to compensate for the lower efficiency of anaerobic glycolysis in producing ATP, these cancer cells demand much higher glucose supply (Warburg, 1956; Vander Heiden et al., 2009). These metabolic characteristics point to the huge demand of cancer cells for carbohydrate substrates, which creates the possibility of treating tumors by exploiting this feature (Patra et al., 2013; DeBerardinis and Chandel, 2016).

Importantly, glucose transporters (GLUTs), particularly GLUT1 and GLUT3, which deliver the carbohydrate substrate in both glycolysis and oxidative phosphorylation pathways, were found to be overexpressed in most cancer cells (Amann et al., 2009; Krzeslak et al., 2012). Consequently, the abnormally high expression of GLUT1 and GLUT3 has been suggested as a considerable factor affecting the deterioration of cancer (Younes et al., 1997; Haber et al., 1998). Based on these rationales, strategies such as ‘starving off the cancers’ have been entertained (Katt et al., 2019), nonetheless, little success has been reported perhaps due to redundancy in live cells. In this report, we consider a novel strategy to slow down the growth and proliferation of tumors by inhibiting glycometabolism using specific small molecule inhibitors of various GLUTs. Such proof-of-concept might validate a new approach for the development of anticancer therapeutics.

In this study, we designed a series of hydantoin derivatives as new inhibitors of GLUT1 or GLUT3 based on the structure of GLUTs (Fig. S1 and Scheme S1). Through the structure-activity relationship study (Table S1 and Fig. S2–5), the compound TH-G313B was found to be an excellent dual inhibitor of GLUT1 and GLUT3, of which the IC_50_s for GLUT1 and GLUT3 in the counterflow assay (protein concentration 20 μmol/L) were determined to be 5.59 ± 1.79 μmol/L and 1.66 ± 0.48 μmol/L, respectively (Fig. 1A). Simultaneously, its inhibitory activity for GLUT1 was similar to cytochalasin B (CB, Table S1), a previously identified inhibitor of GLUT1 used as positive controls.

**Figure 1:**
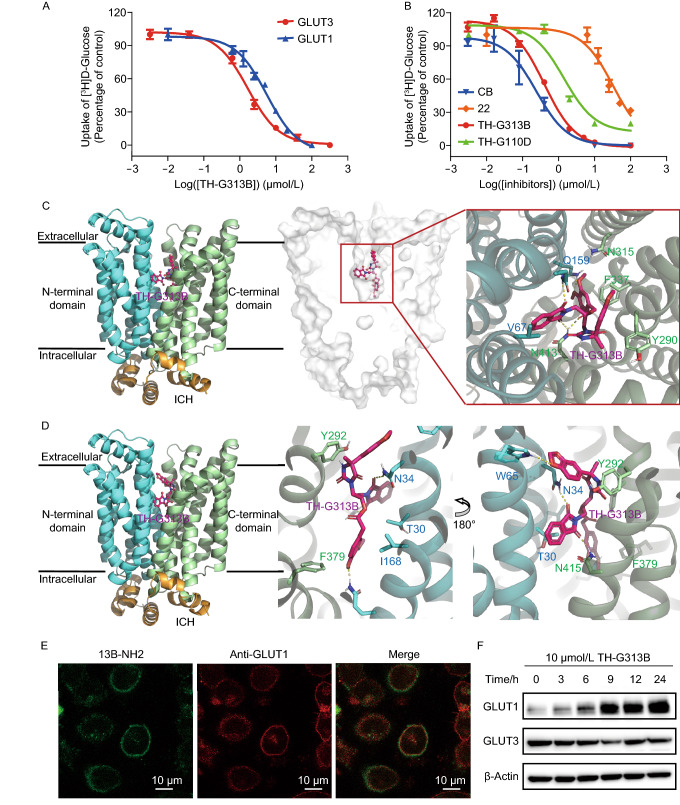
**The interaction between TH-G313B and GLUTs**. (A) The activity of TH-G313B to GLUT1 and GLUT3. The IC_50_ of the TH-G313B for GLUT1 and GLUT3 in the counterflow assay was 5.59 ± 1.79 μmol/L and 1.66 ± 0.48 μmol/L, respectively. (B) Dose-dependent inhibition on GLUT1 in CHO-G1 cells. (C) Analysis of interaction mechanism of GLUT3 and TH-G313B by docking. (D) The interactions between GLUT1 and TH-G313B in docking analysis. (E) Fluorescence imaging of 13B-NH_2_ and anti-GLUT1 in H460 cells. (F) The protein levels of GLUTs were analyzed in H460 cells incubated for 24 h, in which cells were treated with the indicated time of TH-G313B at 10 μmol/L. Data are represented as mean ± SEM

In order to further elucidate the binding mode of the lead compound, TH-G313B, we attempted to co-crystalize TH-G313B with various GLUT family proteins. As integral membrane proteins, these GLUTs are highly challenging targets for X-ray crystallographic analyses. As an alternative, we carried out further computational docking analysis, shedding further insight to the molecular recognition of TH-G313B. Since TH-G313B showed better activity to GLUT3, we studied its binding model with reported GLUT3 structure firstly. With the docking analysis with outward-open GLUT3 (PDB: 4ZWC), it presented several distinct hydrogen bonds of TH-G313B with N32, Q159 and N143 (two hydrogen bonds) residues (Fig. 1C). Meanwhile, we noticed that TH-G313B occupied the glucose-binding pocket of GLUT3, suggesting that TH-G313B could be a competitive inhibitor of glucose. Simultaneously, due to the absence of the structure of GLUT1 in outward-open conformation, we constructed the outward-open GLUT1 model, based on the structure of GLUT3. The calculated binding energy showed that TH-G313B was prefer to bind to the outward-open GLUT1 (Table S2), so we used the outward-open GLUT1 model to analyze the interaction with TH-G313B. In the docking result, it was exhibited that there were also obvious interactions between TH-G313B and residues of GLUT1 (N34, Q161, W65 and N415) (Fig. 1D).

Having identified potent inhibitors of GLUT1 and GLUT3 in biophysical tests, we next tested whether these inhibitors indeed regulate the cancer cells growth by targeting GLUTs. As the first step, on-target validation for TH-G110D and TH-G313B *in vitro* was carried out. In CHO-G1 cells (GLUT1-overexpressed CHO cells), both TH-G110D and TH-G313B were confirmed to evidently inhibit uptake of D-[2-^3^H] glucose in a dose-dependent manner with IC_50_ of 1.43 ± 0.06 μmol/L and 0.40 ± 0.06 μmol/L, respectively (Fig. 1B). The activity of TH-G313B in blocking glucose uptake was significantly higher than that of the original molecule 22, and similar to cytochalasin B (CB, IC_50_ was 0.24 ± 0.07 μmol/L).

Next, we performed further tests in cancerous cells. First, we designed a fluorescent molecule, 13B-NH_2_ by introducing an amino group to the structure of TH-G313B (Fig. S6B). Using fluorescence microscopic imaging, we found that 13B-NH_2_ co-localized with GLUT1 in H460 cells, thereby indicating that these compounds may directly target GLUT1 in cancer cells (Fig. 1E). We also studied the effects of TH-G313B on GLUT1 and GLUT3 in H460 cancer cells. Upon treatment of TH-G313B (10 μmol/L), the level of GLUT1 increased constantly with the over a period of 24 h (Fig. 1F). Under the same condition, the mRNA level of GLUT1 also increased after incubation with 10 μmol/L TH-G313B (Fig. S7). As a comparison, the level of GLUT3 and its mRNA showed negligible changes with the TH-G313B treatment. In general, these results seem to suggest that H460 cells would up-regulate the expression of GLUT1 when its glucose uptake was restricted, possibly due to its role of major transporter for basal glucose, but it was not applicable for GLUT3 (Kurata et al., 1999).

We next probed the effects of TH-G313B on cancer cell growth. We detected the inhibition of TH-G313B on glucose uptake in H460 cells by 2-NBDG, a glucose fluorescent derivative generally used to monitor glucose uptake of cells. The fluorescence signal of 2-NBDG in H460 cells incubated with 10 μmol/L TH-G313B was significantly lower than that of the control cells, indicating that TH-G313B directly inhibited the glucose uptake of H460 cells (Fig. 2A). Next, using the established WST-1 cytotoxicity assay, we found that the proliferation of cancer cells was suppressed by TH-G313B in a dose-dependent manner (Fig. 2B). Upon treatment with 10 μmol/L TH-G313B for 24 h, the apoptosis rate of H460 cells increased significantly relative to the control group, upregulated to the level of that under the low glucose condition (2 mmo/L glucose) (Fig. 2C). However, it was worth noting that TH-G313B was found to have no significant influence to the proliferation of Hek293T cells at various concentrations up to 25 μmol/L (Fig. S6A).

**Figure 2:**
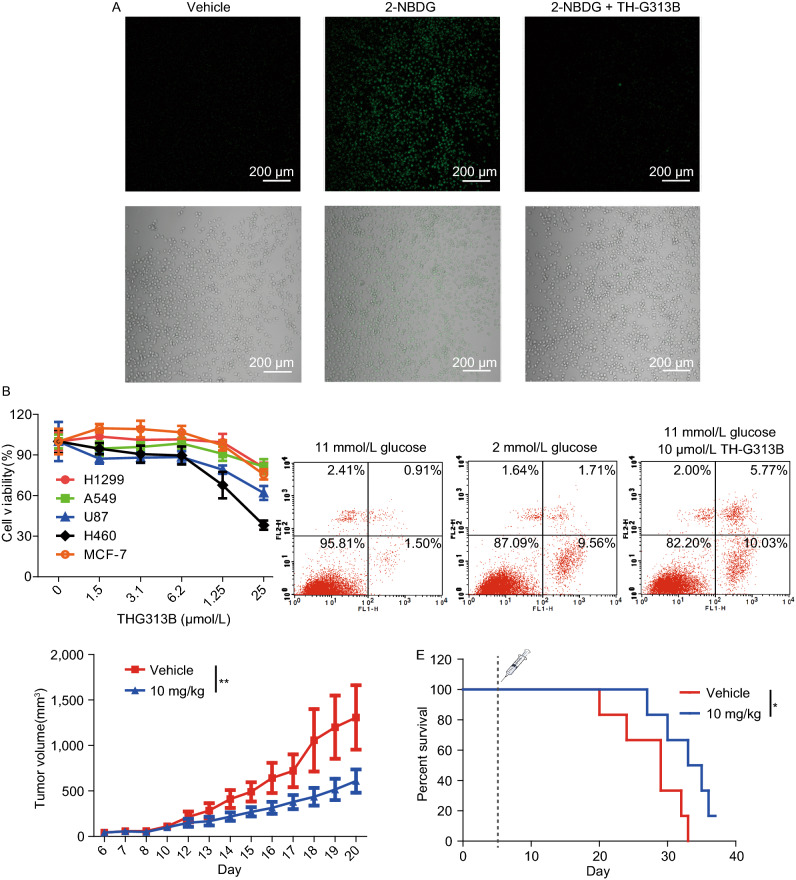

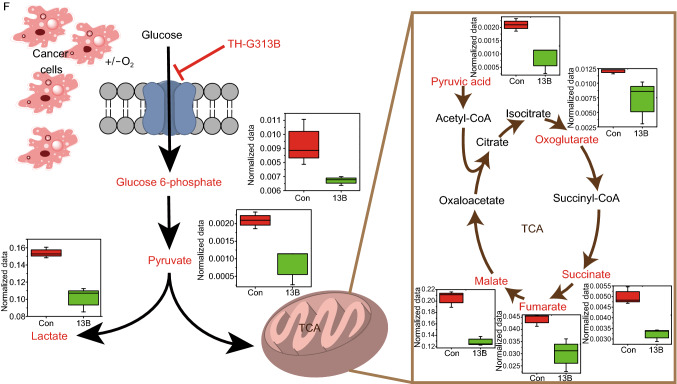
**Inhibitory effects of TH-G313B on cancer cell*in vitro*and*in vivo***. (A) The accumulation of 2-NBDG (50 μmol/L) in H460 cells treated with or without TH-G313B (10 μmol/L) was observed by confocal microscopy (×10). (B) The viability of cancer cells after treated with TH-G313B for 24 h. (C) Apoptosis of H460 cells incubated with normal medium (contained 11 mmo/L glucose), low-glucose medium (contained 2 mmo/L glucose) and TH-G313B (10 μmol/L in normal medium). (D) Tumor volume of H460 tumors in nude mice that received daily subcutaneous dosing of TH-G313B or vehicle (*n* = 6 per group; *P* < 0.005; data represents mean ± SEM). (E) Kaplan–Meier survival analysis of mice received daily subcutaneous dosing of TH-G313B or vehicle starting on Day 6 (*n* = 6 mice per group). *P* < 0.05 Mantel-Cox log-rank test. (F) The metabonomic analysis of glycolysis and TCA cycle in H460 cells incubated with TH-G313B (10 μmol/L) for 24 h, including lactate pathway and TCA cycle (TH-G313B abbreviated to 13B). Data are represented as mean ± SEM

In order to evaluate the inhibitory effects of TH-G313B on cancer cells *in vivo*, we tested whether GLUTs inhibitors suppress the growth of established tumor xenografts in nude mice. Six days after inoculation with H460 cells, nude mice were subcutaneous dosed with or without 10 mg/kg TH-G313B daily. The tumor volume was significantly reduced in the TH-G313B-treated groups relative to the control groups, suggesting that the tumor proliferation was obviously slowed by TH-G313B (Fig. 2D). The mice in vehicle-treated group died from 20th day to 33rd day. In contrast, the mice in the TH-G313B-treated group died from the 27th day, while one mouse survived until the 37th day with a smaller tumor. Overall, TH-G313B treatment increased median survival from 29 to 34 days (Fig. 2E). The tumor volume and survival rate both indicated that TH-G313B could slow down the tumor proliferation and the death of mice.

To further evaluate the effects of TH-G313B on disease burden, we observed the tissue sections of the mice in the vehicle-treated group, the TH-G313B-treated group and the healthy group. The organs showed the same performance in the groups with/without vehicle-treatment, indicating that vehicle was non-toxic to organs. Compared with the healthy mice, the liver tissue of the vehicle-treated group showed blurred nucleus edges, decreased hepatic sinusoids and increased neutrophils. Meanwhile, the renal tubules of the vehicle-treated group became larger with less glomeruli in the kidney section. while its spleen section showed increased lymphocytes. In comparison, for the TH-G313B-treated group, the level of these corresponding changes was similar to the healthy group (Fig. S8). Taken together, these results supported that the tumor proliferation and disease burden were retarded by TH-G313B treatment, which exhibited no additional toxicity to tissues.

Although all of the above data suggested that TH-G313B can impede the proliferation of tumors, it cannot completely cure and eliminate tumors. In an attempt to explain this, we further verified the effects of TH-G313B on the metabolic pathway in H460 cells from the perspective of metabolomics. When we treated H460 cells with 10 μmol/L TH-G313B for 24 h, the intermediates of the tricarboxylic acid cycle (TCA cycle) decreased significantly, such as pyruvic acid, oxoglutarate, succinate, fumarate and malate, as well as the glycolysis pathway (Fig. 2F). However, with further analysis of the metabolites increased significantly, we found several pathways enhanced with TH-G313B treatment (Fig. S9A). The first enriched pathway was the nicotinate and nicotinamide metabolism and some intermediates increased, especially nicotinic acid (Fig. S9B), which was reported to active autophagic flux to protect tumor cells survival against apoptosis (Kim et al., 2016). For the methionine metabolism, we found that its main intermediates increased with TH-G313B treatment, including the cysteine and methionine (Fig. S9B). Methionine is the essential amino acid in one-carbon metabolism and required by cancer cells under cell stress (Wang et al., 2019). Another increased metabolic pathway was purine metabolism (Fig. S9B), thus supplying one-carbon metabolism products as building blocks to promote cancer cells proliferation (Wang et al., 2017). Upon TH-G313B treatment, H460 cells presented obvious increase in lactose degradation, in which lactose and galactose both increased (Fig. S9B). Conversely, the other degradation product, glucose, may be used by cancer cells for survival. Taken together, it suggested that cancer cells upregulated other proliferation-related metabolic pathways to promote its survival under energy stress from the TH-G313B treatment.

The diversity of metabolic pathways in cancer represents a key opportunity for drug development and precision medicine. Due to the rapid proliferation and the special microenvironment, cancer cells are more voracious than normal cells, especially in glucose uptake, to meet the need of energy and material basis. Therefore, we proposed a potential way to inhibit the proliferation of cancer cells by controlling their `feeding’ with the inhibitors of GLUTs. For this purpose, we successfully identified compound TH-G313B as potent inhibitors that specifically blocked glucose uptake mediated by GLUT1 or GLUT3. Notably, compound TH-G313B, with negligible cytotoxicity, was effective in inhibiting GLUT-mediated ‘feeding’ and restricted cancer cell growth both *in vitro* and *in vivo*. These results indicated that GLUTs could be a reliable target in this novel therapeutic strategy for the metabolic reprogrammed tumors. In addition, we noted that inhibiting glycometabolism alone is inadequate for the complete cure of tumors, thus, transporters and metabolic pathways of tumors need to be explored in depth.

## Electronic Supplementary Material

Below is the link to the electronic supplementary material.

(PDF 2239 kb)

## References

[CR1] Amann T, Maegdefrau U, Hartmann A, Agaimy A, Marienhagen J, Weiss TS, Stoeltzing O, Warnecke C, Scholmerich J, Oefner PJ (2009). GLUT1 expression is increased in hepatocellular carcinoma and promotes tumorigenesis. Am J Pathol.

[CR2] DeBerardinis RJ, Chandel NS (2016). Fundamentals of cancer metabolism. Sci Adv.

[CR3] Haber RS, Rathan A, Weiser KR, Pritsker A, Itzkowitz SH, Bodian C, Slater G, Weiss A, Burstein DE (1998). GLUT1 glucose transporter expression in colorectal carcinoma: a marker for poor prognosis. Cancer.

[CR4] Hanahan D, Weinberg RA (2011). Hallmarks of cancer: the next generation. Cell.

[CR5] Katt WP, Lukey MJ, Cerione RA (2019). Starving the devourer: cutting cancer off from its favorite foods. Cell Chem Biol.

[CR6] Kim SW, Lee JH, Moon JH, Nazim UMD, Lee YJ, Seol JW, Hur J, Eo SK, Lee JH, Park SY (2016). Niacin alleviates TRAIL-mediated colon cancer cell death via autophagy flux activation. Oncotarget.

[CR7] Krzeslak A, Wojcik-Krowiranda K, Forma E, Jozwiak P, Romanowicz H, Bienkiewicz A, Brys M (2012). Expression of GLUT1 and GLUT3 glucose transporters in endometrial and breast cancers. Pathol Oncol Res.

[CR8] Kurata T, Oguri T, Isobe T, Ishioka S, Yamakido M (1999). Differential expression of facilitative glucose transporter (GLUT) genes in primary lung cancers and their liver metastases. Jpn J Cancer Res.

[CR9] Patra KC, Wang Q, Bhaskar PT, Miller L, Wang ZB, Wheaton W, Chandel N, Laakso M, Muller WJ, Allen EL (2013). Hexokinase 2 is required for tumor initiation and maintenance and its systemic deletion is therapeutic in mouse models of cancer. Cancer Cell.

[CR10] Pavlova NN, Thompson CB (2016). The emerging hallmarks of cancer metabolism. Cell Metab.

[CR11] Vander Heiden MG, Cantley LC, Thompson CB (2009). Understanding the Warburg effect: the metabolic requirements of cell proliferation. Science.

[CR12] Wang XX, Yang KL, Xie Q, Wu QL, Mack SC, Shi Y, Kim LJY, Prager BC, Flavahan WA, Liu XJ (2017). Purine synthesis promotes maintenance of brain tumor initiating cells in glioma. Nat Neurosci.

[CR13] Wang ZX, Yip LY, Lee JHJ, Wu ZW, Chew HY, Chong PKW, Teo CC, Ang HYK, Peh KLE, Yuan J (2019). Methionine is a metabolic dependency of tumor-initiating cells. Nat Med.

[CR14] Warburg O (1956). On the origin of cancer cells. Science.

[CR15] Younes M, Brown RW, Stephenson M, Gondo M, Cagle PT (1997). Overexpression of GLUT1 and GLUT3 in stage I nonsmall cell lung carcinoma is associated with poor survival. Cancer.

