# Supplementary material for: Regulation of aerobic glycolysis to decelerate tumor proliferation by small molecule inhibitors targeting glucose transporters
Source: Protein Cell. 2020 May 15;11(6):446–51. doi: 10.1007/s13238-020-00725-7 (PMC7251022; doi:10.1007/s13238-020-00725-7)
Supplement: Supplementary file 1 — (PDF 2239 kb) [file 13238_2020_725_MOESM1_ESM.pdf]

Regulation of aerobic glycolysis to decelerate tumor proliferation by small molecule inhibitors targeting glucose transporters

RUNNING TITLE

Decelerate tumor proliferation by GLUTs inhibitors

Meng Gao,[1,2,3] Jian Huang,[1,2,3] Xin Jiang,[2,3,4,5] Yafei Yuan,[2,3,4] Huanhuan Pang,[6] Shuchen Luo,[1,2,3] Nan Wang,[2,3,4] Chengbo Yao,[1,2,3] Zuwan Lin,[1,2,3] Debing Pu,[2,3] Shuo Zhang,[2,3,4] Pengcheng Sun,[2,3,4] Zhuoyi Liu,[1,2,3] Yu Xiao,[7] Qian Wang,[7] Zeping Hu,[6] and Hang Yin[2,3,6,8,*]

[1]Key Laboratory of Bioorganic Phosphorus Chemistry and Chemical Biology (Ministry of Education), Department of Chemistry, Tsinghua University, Beijing 100084, China
[2]Tsinghua University–Peking University Joint Center for Life Sciences, Tsinghua University, Beijing 100084, China.
[3]Beijing Advanced Innovation Center for Structural Biology, Tsinghua University, Beijing 100084, China.
[4]State Key Laboratory of Membrane Biology, School of Life Sciences, Tsinghua University, Beijing 100084, China.
[5]Department of Molecular Biology, Princeton University, Princeton, NJ 08544, USA
[6]School of Pharmaceutical Sciences, Tsinghua University, Beijing 100084, China.
[7]Laboratory Medicine Center, Zhujiang Hospital, Southern Medical University, Guangzhou, Guangdong 510515, China.
[8]Lead Contact
* Correspondence: yin_hang@tsinghua.edu.cn

KEYWORDS

glucose transporter, inhibitor, aerobic glycolysis, cancer cell, proliferation.

Table of Content

1. Supplemental Scheme and Figures

Scheme S1. The synthetic route for the scaffold of hydantoin derivatives.

Figure S1. Identification of hit molecule

Figure S2. The activity of **42** and **TH-G110D** to GLUT1 and GLUT3.

Figure S3. The more SAR study based on the structure of **42**

Figure S4. The activity of inhibitors to GLUT3 and chiral resolution of **51**

Figure S5. The chiral configuration of **TH-G313B**

Figure S6. The influence of **THG313B** to non-cancer cells and identification of **13B-NH$_2$**

Figure S7. The mRNA levels of GLUTs with **TH-G313B** treatment

Figure S8. The tissue sections of the mice with or without treatment of **TH-G313B**

Figure S9. The metabolic pathways enriched according to increased metabolites

Table S1. The structure–activity relationship of the hydantoin analogues for the inhibition of GLUT1

Table S2. The complex energy of GLUT1 in different conformation with **TH-G313B**

2. Supplemental Experimental Procedures

Cell culture

High-throughput virtual screening

hGLUT1 and hGLUT3 protein expression and purification

Counterflow assay

General chemistry methods

Chemical synthesis

ECD calculations.

Homology modelling and molecular docking simulation.

Glucose uptake assay in CHO-G1 cells

Immunoblotting

qRT-PCR analysis

Confocal imaging of colocalization

Imaging of 2-NBDG uptake

Apoptosis assay

WST-1 cytotoxicity assay

Metabolite extraction and metabolomics

Animal experiments

3. Supplemental References

4. Synthesis and Experimental data

1. Supplemental Scheme and Figures

To develop novel small molecule probes, the 1.2 million-compound *Enamine* library was first virtually screened against the β-NG-binding domain of GLUT1 (crystal structure PDB: 4PYP) using the Glide 5.6 program(Halgren et al., 2004). Twenty small molecules were obtained with binding energies below -10 kJ/mol for the consequential counterflow assays. Fortunately, we found four potential hits (Figure S1A) that inhibited GLUT1 activation by at least 45% at 100 μM (Figure S1B). However, in this activity test, if the small molecules have a destructive effect on the vesicle membrane, it will cause a false positive for D-[2-^{3}H]glucose loss. Hence, these small molecules were tested for their inhibitory activity on another transport protein, Adic, which transports arginine. The results suggested that these small molecules not only had no inhibitory effect on Adic (Figure S1C), but also had no destructive effect on the vesicle membrane, thereby eliminating the false positive. Eventually, upon evaluation of the activity and selectivity between GLUT1 and GLUT3, compound **18**, a novel molecule containing hydantoin and phthalimide structures, was identified as the hit molecule for further SAR study. (Figure S1D).

Based on the promising preliminary results, we attempted to optimize the hit molecule **18** with structure-activity relationship studies. Thus, we developed a general synthetic route (Scheme S1) and designed a series of analogues to explore the pharmacophore of **18** (Table S1). We used a previously established biophysical counterflow assay to determine their inhibitory activities against GLUT1 and GLUT3, because only single target protein in this system was conducive to directly test the inhibition of molecules. (Table S1 and Figure S4A). Furthermore, we also investigated the effects of stereogenic center at the C13 position by stereo-selectively preparing both R- and S-isomers.

Firstly, when we replaced the phenyl group with the butyl group at the R_1 position in **21/22**, the activity still retained (**27/28**). Then, we found that the conversion of the butyl group at R_2 to phenyl group (**23/24**) decreased the activity significantly. Correspondingly, the conversion to methyl groups (**25/26**) had little effect on the activity. Interestingly, introducing substituent groups to phenyl ring had little impact on activity, including electron-withdrawing groups (**37/38/39/40**) and electron-donating groups (**41/42/43/44**). Accordingly, the chemical modifications of R_1 and R_2 led to no significant change in activity, except for introduction of rigid and bulky groups at both R_1 and R_2 position.

The SAR study for R_3 position indicated that phthalimide was critical for activity. The conversion of the phthalimide decreased the activity whether to indole or carbazole (**31/32/35/36**). Meanwhile, removing the phenyl ring of phthalimide resulted in significant decrease of inhibition (**29/30**), as well as hydrogenating the phenyl ring (**33/34**). Thus, in the following SAR studies, the phthalimide group was fixed at the R_3 position.

Based on these results, compound **42** was chosen as the structural basis for further optimization due to its higher activity than other analogues, especially its diastereomer **41**. By introducing the aromatic group linked by alkyl ester at the R_4 position, we found that compound **TH-G110D** (**45**) significantly improved the activity with a selectivity of GLUT1 over GLUT3 (Figure S2). Finally, when we shortened the ester chain of **TH-G110D**, compound **51** was obtained and displayed the highest inhibitory activity for both GLUT1 and GLUT3.

In order to evaluate the key active sites of **51**, we designed several molecules with two ring structure (Figure S3). However, these molecules showed lower activity, confirming that all the three rings contributed to its activity. Furthermore, chiral resolution and configuration identification of **51** were conducted. Upon separation of the reaction products of **51** by chiral HPLC, two components with M/z 638.17352 (the peaks numbered 3 and 5) were found to be the two diastereomers of **51**, in which **51-3** (named by **TH-G313B**) showed the best activity to GLUT1 and GLUT3 in the counterflow assay (Figure S4C). However, **TH-G313B** was too flexible to obtain its crystal structure, its chiral configuration was assumed to be SR by comparing its experimental circular dichroism spectra and the calculated spectra (Figure S5).

Combined with the docking results, it was speculated that the benzene ring of the inhibitor at R_3 fitted into the adjacent cavity pocket, as well as the pocket for R_1 and R_2, which was incompatible with diphenyl group. In addition, the interactions between the phthalimide with N34 and N415 were indispensable because replacing phthalimide with other substituents decreased activity substantially. Moreover, the hydrogen bond between Q161 and para-methoxyl at R_4 enhanced the inhibitory activity of **TH-G313B**. Finally, the ester chain of **TH-G313B** was stabilized by hydrogen bonding with the residues N413 (Figure 1C and 1D).

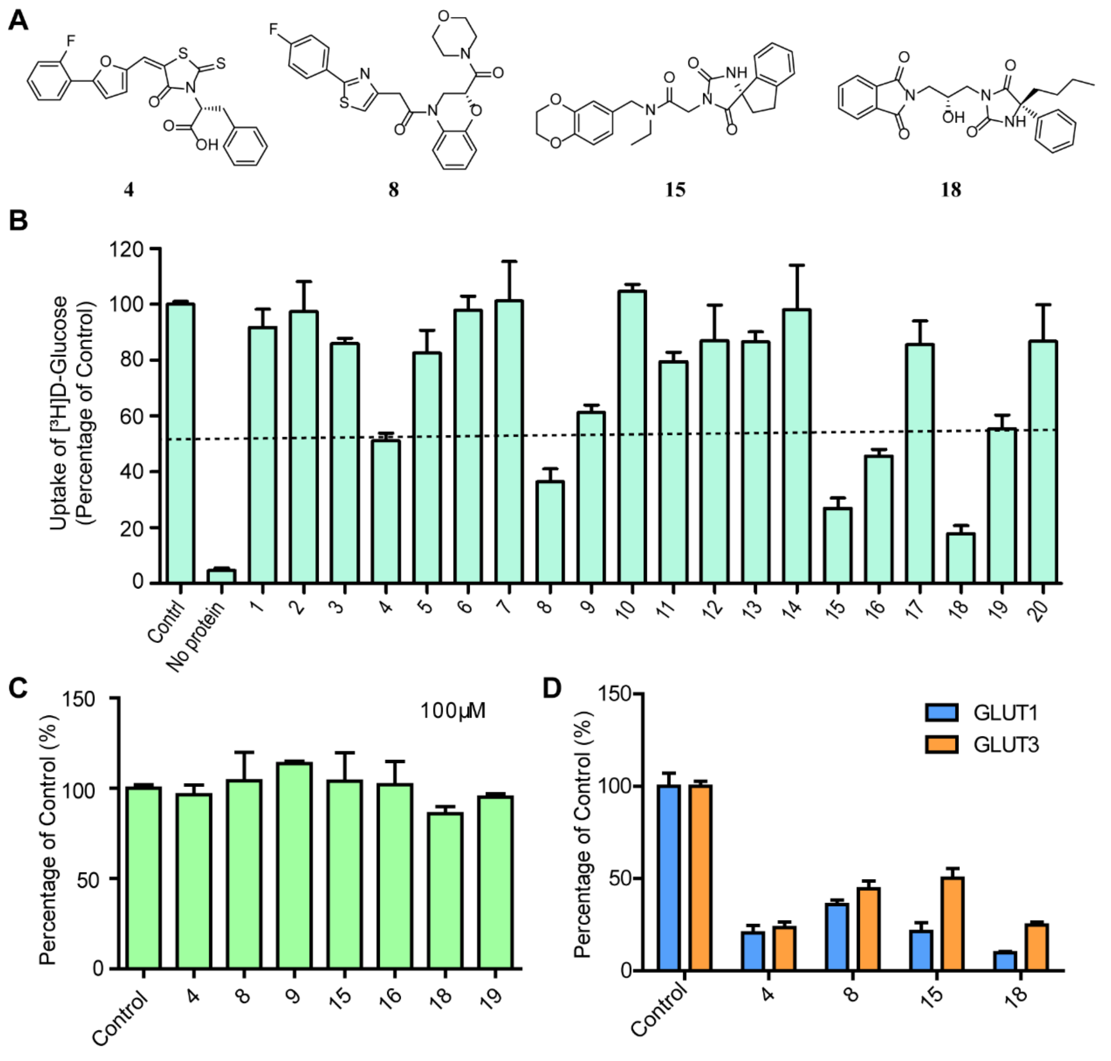

Figure S1. Identification of hit molecule.

(A) The structure of **4**, **8**, **15**, and **18**.

(B) The inhibitory activity of 20 small molecules on the GLUT1 at 100 μM in counterflow assay.

(C) The inhibitory activity on Acid.

(D) The selectivity of **4**, **8**, **15**, and **18** to GLUT1 and GLUT3 at 100 μM. Data are represented as mean ± SEM.

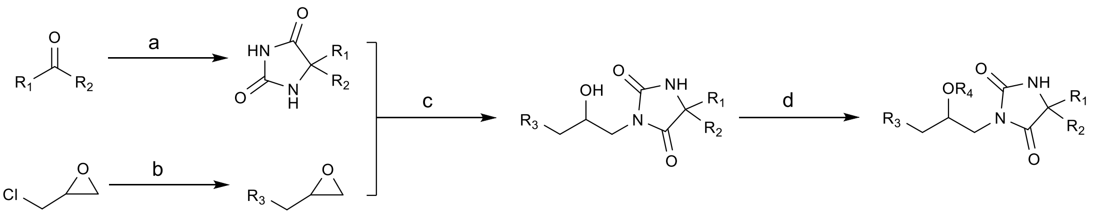

Scheme S1. The synthetic route for the scaffold of hydantoin derivatives. (a) KCN (2.0 equiv.), $(NH_4)_2CO_3$ (4.0 equiv.), 50 % EtOH/H_2O, 60 °C, 12 h. (b) nucleophilic reagent (0.25 equiv.), DMF, 120 °C, 2 h. (c) base (KOH or K_2CO_3, 2.5-3 equiv.), DMF, 120 °C, 3 h. (d) carboxylic acids (1.2 equiv), DMAP (0.12 equiv), EDCI (1.5 equiv), dry DCM, r.t., 12h.

Table S1. The structure–activity relationship of the hydantoin analogues for the inhibition of GLUT1.

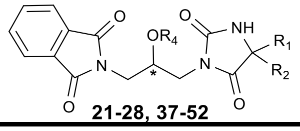

21-28, 37-52

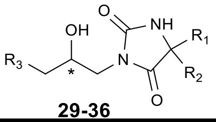

29-36

Cpd	*	R_1	R_2	R_3	R_4	Inhibition[a]
**21**	R	-Ph	$-CH_2(CH_2)_2CH_3$		H	77.0 ± 2.8
**22**	S					76.1 ± 4.7
**23**	S	-Ph	-Ph		H	13.0 ± 4.6
**24**	R					25.0 ± 10.2
**25**	S	-Ph	$-CH_3$		H	54.5 ± 1.2
**26**	R					60.7 ± 1.9
**27**	R	$-CH_2(CH_2)_2CH_3$	$-CH_2(CH_2)_2CH_3$		H	80.6 ± 0.9
**28**	S					83.2 ± 4.5
**29**	R	-Ph	$-CH_2(CH_2)_2CH_3$	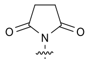	H	< 1
**30**	S					23.7 ± 5.6
**31**	R	$-CH_2(CH_2)_2CH_3$	$-CH_2(CH_2)_2CH_3$	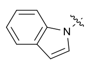	H	44.1 ± 4.8
**32**	S					23.3 ± 7.0
**33**	R	-Ph	$-CH_2(CH_2)_2CH_3$	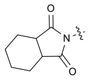	H	< 1
**34**	S					19.4 ± 3.7
**35**	R	$-CH_2(CH_2)_2CH_3$	$-CH_2(CH_2)_2CH_3$	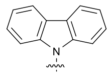	H	42.0 ± 3.2
**36**	S					47.5 ± 5.8
**37**	R	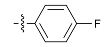	$-CH_2CH_3$		H	59.5 ± 5.4
**38**	S					73.1 ± 2.5
**39**	R	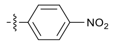	$-CH_3$		H	59.7 ± 0.1
**40**	S					71.3 ± 3.9
**41**	R	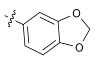	$-CH_3$		H	57.5 ± 2.8
**42**	S					74.5 ± 1.2
**43**	R	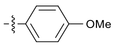	$-CH_3$		H	65.0 ± 2.4
**44**	S					68.0 ± 3.4
**45**	S	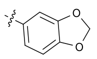	$-CH_3$		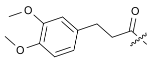	83.7 ± 4.1
**46**	S	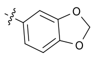	$-CH_3$		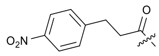	5.4 ± 2.0
**47**	S	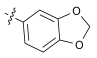	$-CH_3$		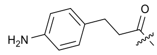	31.3 ± 2.0
**48**	S	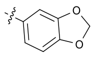	$-CH_3$		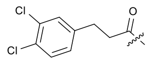	< 1
**49**	S	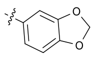	$-CH_3$		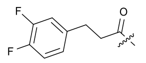	< 1
**50**	S	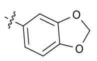	$-CH_3$		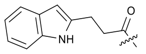	< 1
**51**	S	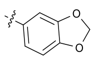	$-CH_3$		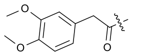	65.9 ± 6.9 [b]
**52**	S	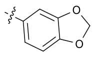	$-CH_3$		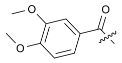	35.8 ± 5.9 [b]
**cytochalasin B**						50.8 ± 3.5[c]

Inhibiton ratio was determined at 100 μM[a], 10 μM[b] and 5 μM[c]. Data are represented as mean ± SEM

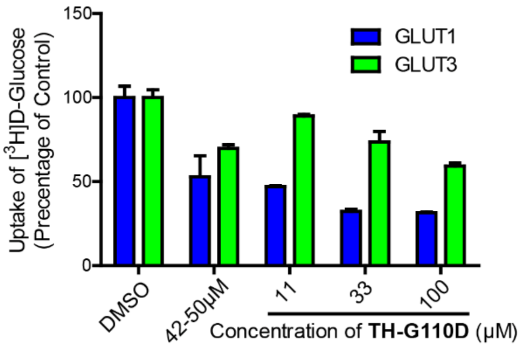

Figure S2. The activity of 42 and TH-G110D to GLUT1 and GLUT3. The activity of 42 and selectivity of TH-G110D between GLUT1 and GLUT3 in counterflow assay.

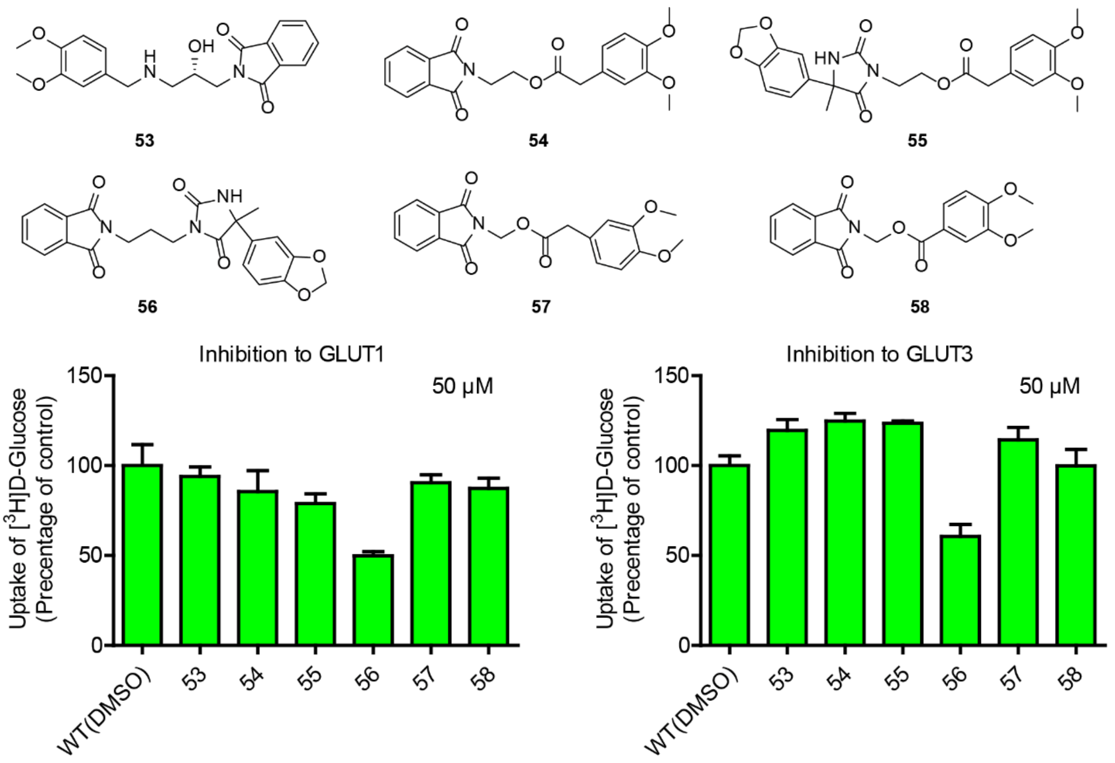

Figure S3. The more SAR study based on the structure of 42. The structure and activity of double-fragment molecules. Data are represented as mean ± SEM.

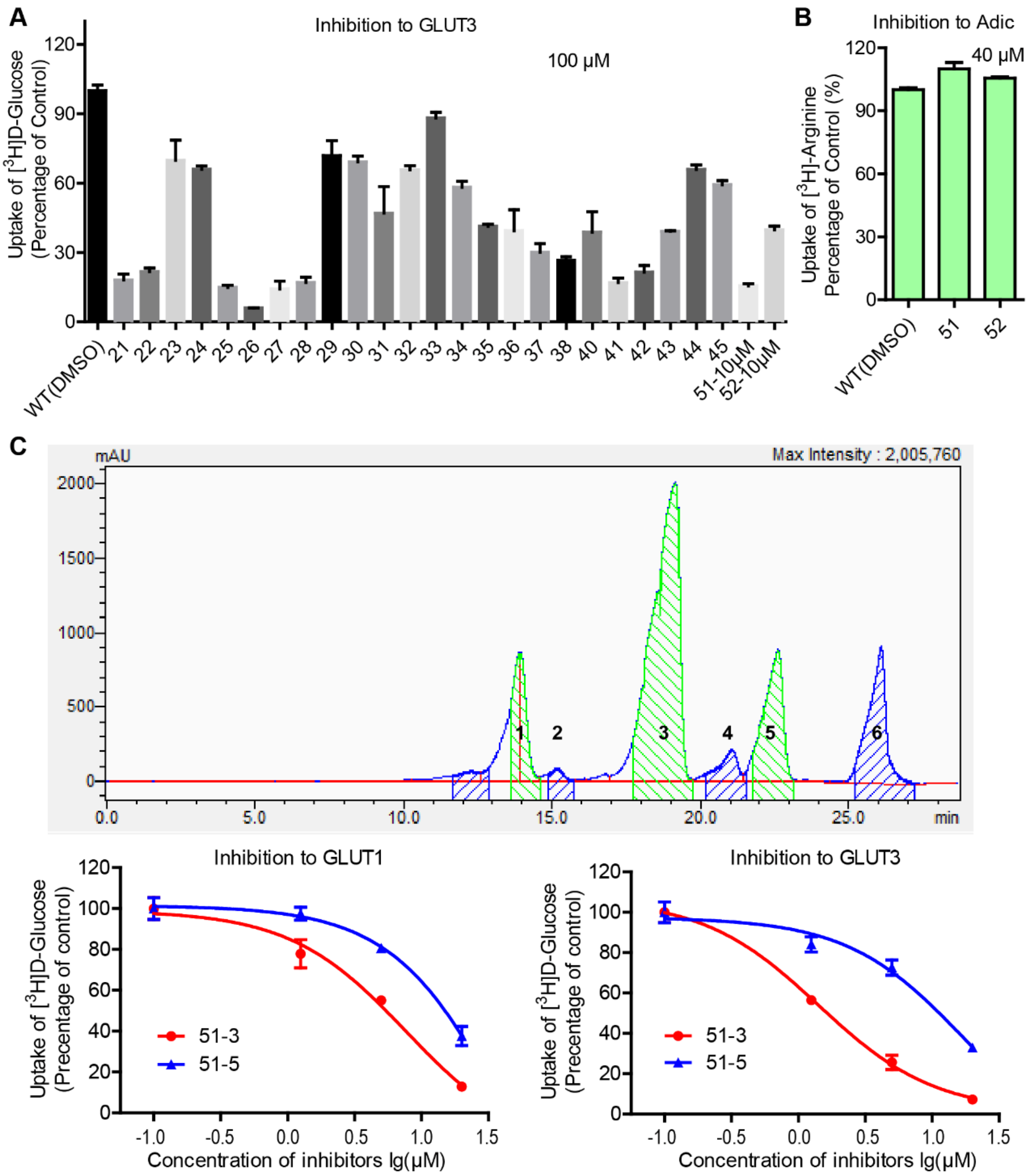

Figure S4. The activity of inhibitors to GLUT3 and chiral resolution of 51.

(A) The inhibition of hydantoin derivatives (100 μM) on GLUT3 at 100μM.

(B) The activity of **51** and **52** to Adic.

(C) Chiral resolution of **51** and the inhibition to GLUT1 and GLUT3. Among them, **51-3** and **51-5** correspond to peaks labeled 3 and 5 in the chromatogram, respectively. Data are represented as mean ± SEM.

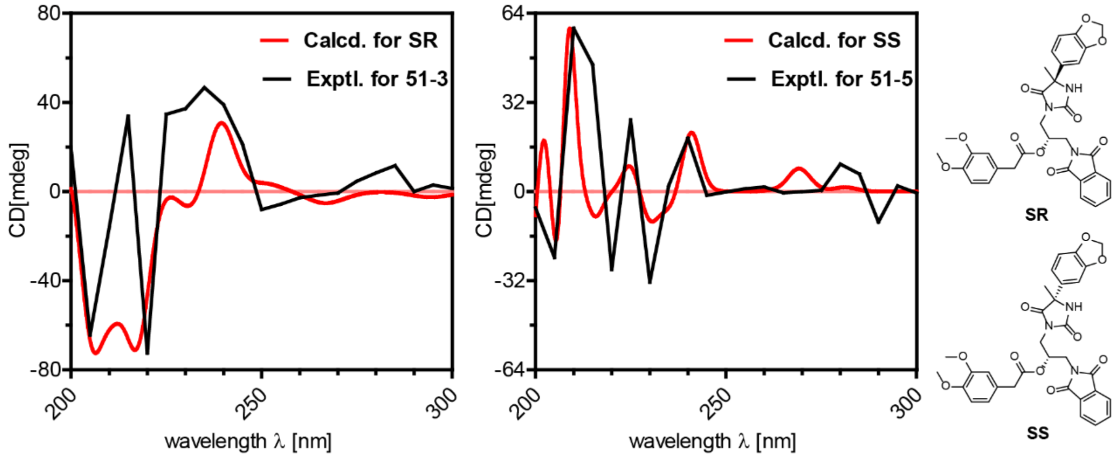

Figure S5. The chiral configuration of TH-G313B. The circular dichroism (CD) of **51-3** and **51-5**, comparing with simulated SS/SR spectra to infer the corresponding configuration.

Table S2. The complex energy of GLUT1 in different conformation with TH-G313B

Conformation of GLUT1	Prime MM-GBSA complex energy (ΔG bind)
Inward-open	-61.2 ± 1.6 [a]
Outward-open	-69.7 ± 1.7 [a]

[a]20 poses generated in total. Data are represented as mean ± SEM.

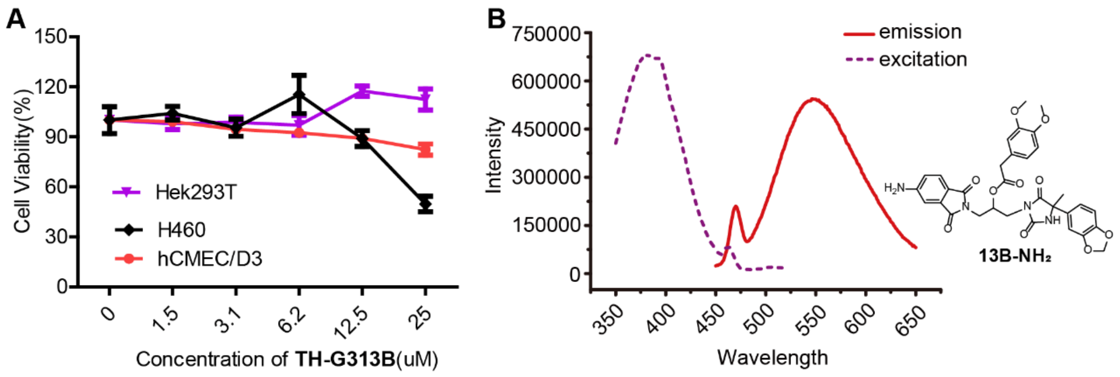

Figure S6. The influence of THG313B to non-cancer cells and identification of 13B-NH$_2$. (A) The viability of Hek293T cells, hCMEC/D3 cells and H460 cells with **TH-G313B** treatment for 40 h.

(B) The structure and fluorescence spectrum of 13B-NH$_2$. Data are represented as mean ± SEM.

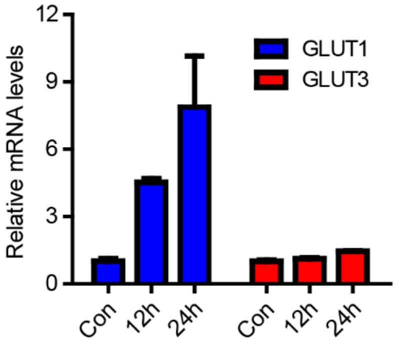

Figure S7. The mRNA levels of GLUTs with TH-G313B treatment. The mRNA levels of GLUTs were analyzed in H460 cells incubated with the indicated time of **TH-G313B** at 10μM.

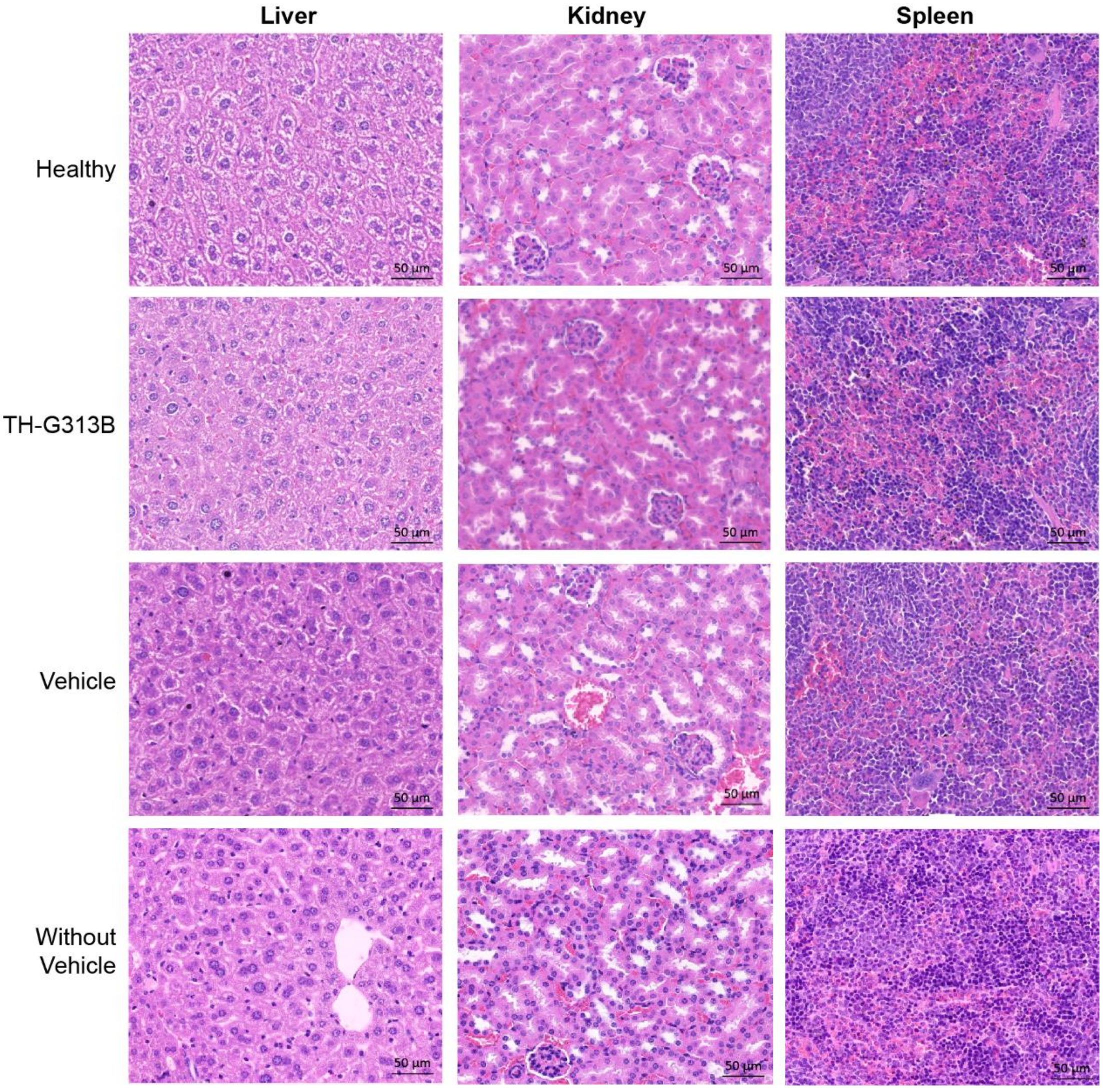

Figure S8. The tissue sections of the mice with or without treatment of TH-G313B. Representative photomicrographs of liver, kidney and spleen of the mice in group with **TH-G313B**-treatment or with/without vehicle-treatment, compared with healthy group (n = 3).

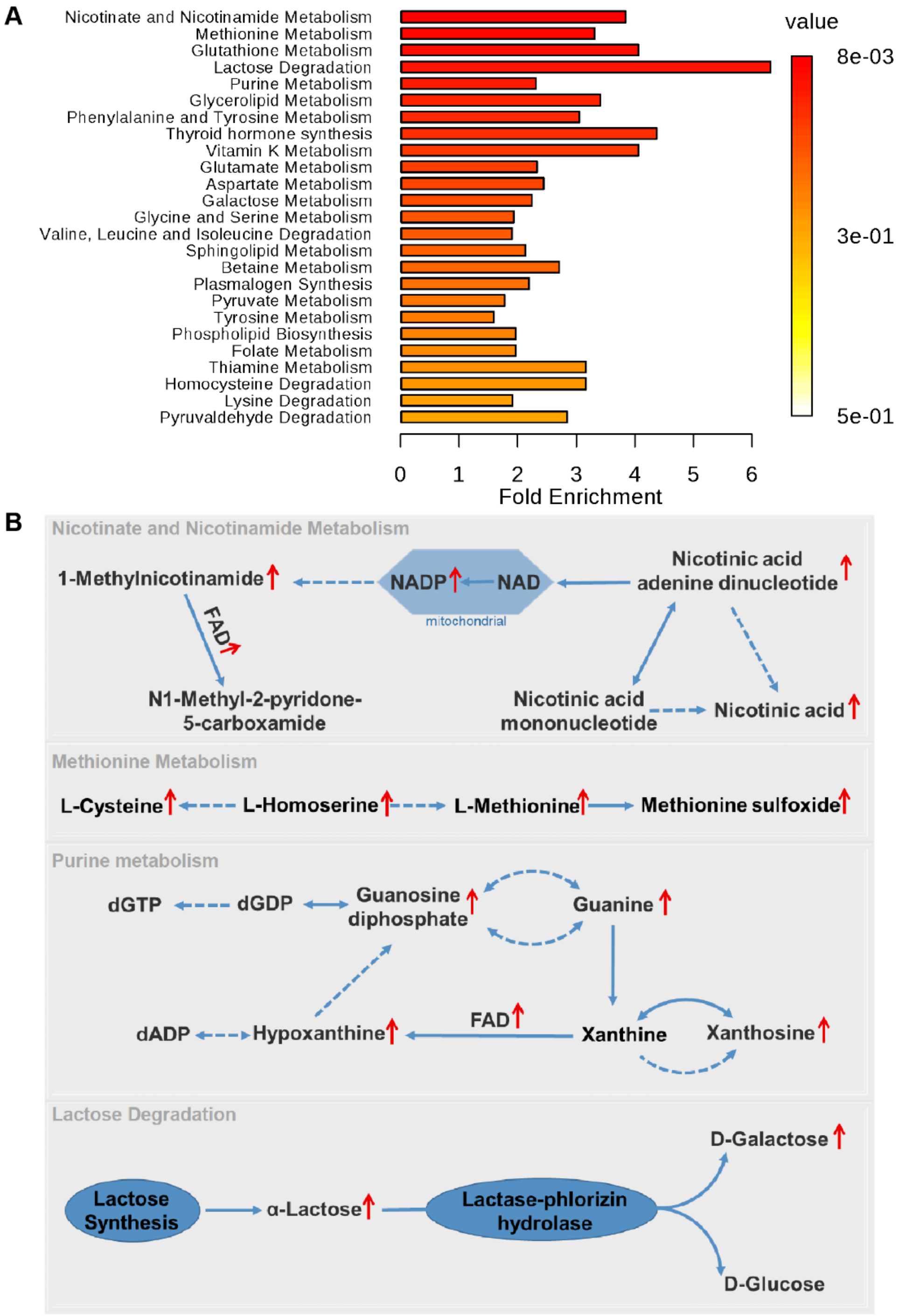

Figure S9. The metabolic pathways enriched according to increased metabolites.

(A) The metabolic pathways enriched according to increased metabolites of H460 cells with **TH-G313B** treatment.

(B) The metabolites obviously increased in the enriched metabolisms of H460 cells.

2. Supplemental Experimental Procedures

Cell culture

The stably expressed hGLUT1 CHO cell line (CHO-G1) was provided by Chen group(Ung et al., 2016). The other cells were purchased from National Infrastructure of Cell Line Resource and were not further authenticated. All cultured cells were grown at 37 °C in a humidified incubator containing 5% CO_2. CHO-G1 cells were cultured in HAM's F12 containing 10% (v/v) FBS, 100 U/mL penicillin, 100 μg/mL streptomycin and 500μg/mL hygromycin B. Hek293T, A549, H1299 and U87MG cells were cultured in Dulbecco's modified Eagle's medium (DMEM), 10% (v/v) FBS, 100 U/mL penicillin and 100 mg/mL streptomycin. H460 and MCF-7 cells were cultured in Roswell Park Memorial Institute (RPMI) 1640 medium supplemented with 10% (v/v) FBS, 100 μg/mL streptomycin and 100 U/mL penicillin. The hCMEC/D3 cells were cultured in ECM medium, 5% (v/v) FBS, 5% (v/v) ECGS,100 U/mL penicillin and 100 mg/mL streptomycin. The cultures were checked periodically and found to be free of mycoplasma contamination.

High-throughput virtual screening

The *Enamine* drug database (1.2 million small molecules) was docked into the β-NG-binding domain of human GLUT1 (PDB: 4PYP) by Glide 5.6. The molecules were created, as appropriate, with multiple protonation and tautomeric states. The human GLUT1 conformations were prepared using standard Glide protocols. This includes addition of hydrogen, restrained energy-minimizations of the protein structure with the Optimized Potentials for Liquid Simulations-All Atom (OPLS-AA) force field, and finally setting up the Glide grids using the Protein and Ligand Preparation Module. All 1.2 million compounds were first docked and ranked using High-Throughput Virtual Screening (HTVS) Glide, continued with standard precision (SP) Glide for the top 10000 compounds. The resultant top 5000 compounds were then docked using the more accurate and computationally intensive extra-precision (XP) mode. Initial top-ranked 100 compounds were selected and ranked by predicted binding energy.

hGLUT1 and hGLUT3 protein expression and purification

The human glucose transporter GLUT1 and GLUT3 are expressed and purified following the protocols that have been described previously(Deng et al., 2015; Deng et al., 2014). Specifically, the synthesized full-length human GLUT1 and GLUT3 complementary DNA were subcloned into a modified pFastbac1 (Invitrogen) vector containing a C-terminal or N-terminal 10x His tag, respectively. To increase the homogeneity of recombinant protein, N45T mutation was introduced into GLUT1 to eliminate the glycosylation. N43T mutation was introduced into GLUT3 for the same purpose. The recombinant GLUT1 and GLUT3 were expressed in the pFastBac baculovirus system (Invitrogen). In Brief, the Bacmid DNAs were generated through the transfection of DH10bac cells with recombinant pFastbac1 vector. Sf9 insect cells (Invitrogen) were used to generate and amplify the baculoviruses. The third generation baculoviruses were applied to the infection of Sf9 cells, which led to the expression of recombinant protein. The Sf9 cells were harvested 3 days later since the infection of baculoviruses. Cells were resuspended and homogenized with

buffer containing 25 mM Tris pH 8.0 and 150 mM NaCl. Protease inhibitors (aprotinin at 5 μg ml^{-1}, pepstatin at 1 μg ml^{-1}, and leupeptin at 5 μg ml^{-1}; Amresco) were added into the cell resuspension to prevent protein degradation. Then, the cell resuspension was flash frozen in liquid nitrogen and stored in -80 ℃ fridge before further purification.

The first step to purify the GLUTs was to extract the recombinant protein from cell membrane with 2% (w/v) *n*-dodecyl-β-D-maltopyranoside (DDM, Anatrace) at 4 °C for 1 h. The debris of cell membrane were removed through centrifugation at 18,700 g for 30 min. The supernatant was applied to Ni^{2+}-nitrilotriacetate affinity chromatography (Ni-NTA, Qiagen) at 4 °C cold room. After the resin was rinsed with washing buffer (25 mM MES pH 6.0, 150 mM NaCl, 30 mM imidazole, and 0.02% (w/v) DDM) to remove the non-specific protein binding, the recombinant protein was eluted with elution buffer that containing 25 mM MES pH 6.0, 150 mM NaCl, 300 mM imidazole, and 0.02% (w/v) DDM. The elution was concentrated to about 1~2 ml and applied to size-exclusion chromatography (Superdex-200; GE Healthcare) for further purification. A selected buffer was applied to each batch of size-exclusion chromatography for specific assay. The peak fractions were collected and flash frozen in liquid nitrogen followed by storage in -80 ℃ fridge before further biochemical analysis.

Counterflow assay

Liposomes were prepared as described previously(Deng et al., 2014). For each assay, 2 μl of concentrated proteoliposomes (GLUT1 or GLUT3) in 96 μl KPM6.5 buffer were incubated with or without inhibitors for 30 min at 0 ºC. Upon added 1 μCi D-[2-^{3}H]glucose (specific radioactivity 21.5 Ci/mmol, PerkinElmer), the mixture was incubated for 30 s and rapidly filtered through 0.22 μm filters (Millipore), following washed by 2 ml ice-cold KPM 6.5 buffer immediately. Finally, the filters were solubilized with 0.5 ml Optiphase HISAFE 3 (PerkinElmer) and used for liquid scintillation counting with MicroBeta JET (PerkinElmer). The positive and negative control were the liposomes with and without protein, respectively.

General chemistry methods

NMR spectra were acquired on a Bruker 400 spectrometer, running at 400 MHz for ^{1}H and 101 MHz for ^{13}C, respectively. ^{1}H NMR spectra were recorded at 400 MHz in $CHCl_3$-*d* and $(CH_3)_2SO$-*d6* using residual $CHCl_3$-*d* (7.28 ppm) and DMSO (2.50 ppm) as an internal reference. ^{13}C NMR spectra were recorded at 101 MHz in $CHCl_3$-*d* and $(CH_3)_2SO$-*d6* using residual $CHCl_3$ (77.16 ppm) and DMSO (39.52 ppm) as an internal reference. Compounds were purified using flash chromatography (silica gel 60Å, 230-400 mesh, Sorbent Tech.). Mass spectrometry was performed at the mass spectrometry facility (Thermo, Q-Exactive) of the Center of Pharmaceutical Technology at Tsinghua University. All commercially reagents were purchased from Sigma-Aldrich, Fisher Scientific, Invivogen, etc. Unless otherwise noted, analytical grade solvents and commercially available reagents were used without further purification. All tested compounds were purified by HPLC (Agilent Technologies 1200 serials) using UV detector at 254 nm and 280 nm.

Chemical synthesis

The general methods for synthesizing the inhibitors are showed in **Scheme S1**, and following procedures takes **51** as example (**Method A**). And the different chemical synthesis procedures of other compounds are described in the **Synthesis and Experimental data**.

5-(benzo[d][1,3]dioxol-5-yl)-5-methylimidazolidine-2,4-dione

To a stirred solution of 1-(benzo[*d*][1,3]dioxol-5-yl)ethan-1-one (821 mg, 5 mmol) in EtOH (23 mL) was added KCN (650 mg in water, 10 mmol, produced by TMSCN and KOH), $(NH_4)_2CO_3$ (1.922 g, 20 mmol) and water (23 mL) at room temperature. The mixture was stirred at 60 °C for 12 h. After the solution was cooled to room temperature, the hydantoin precipitate was filtered, and the filtrate was acidified (pH = 2) by the addition of concentrated HCl to give more precipitate, which was filtered again. The filtrate was concentrated to half-volume and cooled, and the hydantion product that precipitated was filtered. The solids were combined and recrystallized from hot ethanol to give the final product (745 mg, yield = 63.6%).

(S)-2-(oxiran-2-ylmethyl)isoindoline-1,3-dione

To a stirred solution of potassium phthalimide (2g, 10.81 mmol) in DMF (N,N-dimethyl-formamide) (15 mL) was added (*S*)-epichlorohydrin (4g, 43.24 mmol). The mixture was stirred at 120 °C for 2 h. The reaction mixture was diluted in ethyl acetate (30ml), washed with water (50 mL), saturated $NaHCO_3$ (50 mL) and brine (50 mL). After drying with anhydrous Na_2SO_4, the solvent was evaporated in vacuo. The residue was purified by column chromatography to give the final product as white solid (2.01g, yield = 92%).

*2-((2S)-3-(4-(benzo[d][1,3]dioxol-5-yl)-4-methyl-2,5-dioxoimidazolidin-1-yl)-2-hydroxypropyl)isoindoline-1,3-dione (**42**)*

To a stirred solution of *5-(benzo[d][1,3]dioxol-5-yl)-5-methylimidazolidine-2,4-dion* (300 mg, 1.41 mmol) and potassium hydroxide (95 mg, 1.69 mmol) in DMF (10 mL) was added *(S)-2-(oxiran-2-ylmethyl)isoindoline-1,3-dione* (861 mg, 4.24 mmol). The mixture was stirred at 120 °C for 3 h. The reaction mixture was diluted in ethyl acetate (30ml), washed with water (50 mL), saturated $NaHCO_3$ (50 mL) and brine (50 mL). After drying with anhydrous Na_2SO_4, the solvent was evaporated in vacuo. The residue was purified by column chromatography to give the final product as white solid (407 mg, yield = 69%).

*(S)-1-(4-(benzo[d][1,3]dioxol-5-yl)-4-methyl-2,5-dioxoimidazolidin-1-yl)-3-(1,3-dioxoisoindolin-2-yl)propan-2-yl 2-(3,4-dimethoxyphenyl)acetate (**51**)*

To a stirred solution of ***42*** (200 mg, 0.46 mmol) and 3,4-Dimethoxyphenyl acetic acid (108 mg, 0.55 mmol) in dry DCM (10 mL) was added DMAP (7.4 mg, 0.06 mmol) and EDCI (133 mg, 0.69 mmol). The mixture was stirred at r.t. for 12 h. The reaction mixture was evaporated in vacuo. The residue was purified by column chromatography to give the final product as white solid (177 mg, yield = 63%). This crude product will be chirally separated by HPLC (65% MeCN in H_2O), and it is interesting to note that the mixture of **42** and **51** can help **51** to separate chirality better.

ECD calculations

All theoretical calculations were carried out using the Gaussian 09 program package(Frisch MJT GW). Conformation search by MMFF94S molecular force fields were performed for all possible isomers, providing corresponding stable conformers with distributions higher

than 1%(Goto and Osawa, 1993). The conformers were optimized using the DFT method at the B3LYP/6-31+G(d,p) level in gas. Then further conformer analyses were performed according to the frequency and Boltzmann distribution theory to remove unreasonable and unstable conformers, while room-temperature equilibrium proportion were calculated according to the Boltzmann distribution law.

The theoretically calculated ECD spectra were established using the TDDFT method at the B3LYP/6-311+G(d,p) level in dichloromethane, using the PCM model, including in all cases 30 excited states. The ECD spectra were simulated by SpecDis (version 1.64) using σ = 0.15~ 0.30 eV and $R^{velocity}$. All calculated curves were shifted from -30 to +30 nm to better simulate experimental spectra.

Homology modelling and molecular docking simulation

The full protein sequences of human GLUT1 and GLUT3 were obtained from Uniprot(Bateman et al., 2019) and aligned with the multiple sequence alignment program MUSCLE(Chojnacki et al., 2017). Using the alignment results and the crystal structure of outward-open GLUT3 (PDB: 4ZWC) as input, we built 100 homology models of human GLUT1 in outward-open conformation with the software MODELLER(Sali and Blundell, 1993), and the best model was chosen by PROCHECK(Laskowski et al., 1993).

Compound **TH-G313B** was drawn in 2D sketcher in Schrodinger suite and 3D structure was carried out by LigPrep(Schrödinger_Release, 2018-1). All settings were as default except retaining specified chiralities. Protein preparation also used default settings by protein preparation wizard in Maestro. **TH-G313B** was docked against outward-open GLUT3 (PDB: 4ZWC), inward-open GLUT1 (PDB: 4PYP) and outward-open GLUT1 model. Ligand docking was performed by XP glide docking program in schrodinger suite, using the receptor grid and ligand generated as mentioned above.

In order to identify the proper conformation of GLUT1, we firstly used 20 poses per ligand for XP glide docking against inward-open GLUT1 (PDB: 4PYP) and outward-open GLUT1 model. And the binding free energies of all different poses from XP docking outputs were carried out by using Prime-MMGBSA. Based on the binding free energies, we chosen outward-open GLUT1 model as the receptor structure. Then we kept 20000 poses per ligand for the initial phase of docking to identify the proper binding mode, and keep best 2000 poses per ligand for energy minimization for "selection of initial poses". All the molecular graphics were performed with PyMOL.

Glucose uptake assay in CHO-G1 cells

The inhibitory activity of compounds in glucose transport was also analyzed by measuring the uptake of D-[2-^{3}H]glucose in CHO-G1 cells. Cells grown in 24-well plates were washed twice with serum-free MEM and incubated with 0.5 ml of the same medium at 37 °C for 2 h. The cells were washed three times with phosphate buffered saline (PBS) buffer and incubated with 0.45 ml PBS buffer at 37 °C for 30 min. Compounds were then added and incubated for 8 min following with the addition of 0.3 μCi D-[2-^{3}H]glucose (specific radioactivity 21.5 Ci/mmol, PerkinElmer). The known hGLUT1 inhibitor cytochalasin B (10 μM) was positive control. After 8 min, glucose uptake was terminated by washing the cells three times with cold PBS and lysing cells with 0.1 N NaOH. After mixed with 2.5 ml

Optiphase HISAFE 3 (PerkinElmer), the radioactivity retained by the cell lysates was measured by a LS 6000 Series Liquid Scintillation Counter (Beckman Coulter, Inc. Fullerton, CA).

Immunoblotting

Western blot analysis was performed in H460 cells (RPMI1640 medium supplemented with 2% (v/v) FBS, 100 μg/mL streptomycin and 100 U/mL penicillin) treated without/with 10 μM **TH-G313B** for different time in 24h to determine the expression level of human GLUT1 and GLUT3. The cells were washed three times with PBS, and were lysed with the lysis buffer. After being transferred to centrifuge tubes, cell lysates were sonicated and then centrifuged at 12,000 rpm for 15 min. The resulting supernatant was collected and measured for its protein concentration. After protein concentration measurement by Pierce bicinchoninic acid (BCA) protein assay (Pierce Biotechnology, Inc. Rockford, IL), the protein samples were run on 10% Bio-Tris gel (Bio-Rad, Hercules, CA) and transferred to a nitrocellulose membrane (Bio-Rad) by electroblotting (100 mA for 1 h) and probed with the primary antibody human GLUT1 (Abcam, ab15309) at 1:1000 dilution or antibody human GLUT3 (Abcam, ab191071) at 1:1000 dilution. Peroxidase-conjugated AffiniPure Goat Anti-Rabbit IgG (H+L) antibody at 1:3000 dilution were used as secondary antibody. 5% w/v BSA in TBST was used for blocking the membrane, and primary, secondary antibody preparation steps. Visualization of the blots was performed by Thermo SuperSignal West Pico kit (Thermo Fisher Scientific) or by Immobilon Western (Millipore). The internal control was β-actin (Cell Signaling Technology, Inc.).

qRT-PCR analysis

H460 cells were seeded in a 6-well plate with 6 × 10^5 cells per well and incubated in the presence or absence of 10 μM **TH-G313B** in RPMI1640 medium (supplemented with 2% (v/v) FBS, 100 μg/mL streptomycin and 100 U/mL penicillin) for different time, but total incubation time was 24h. Then, cells were washed with PBS and extracted RNA by E.Z.N.A. total RNA Kit (OMEGA), following by reverse transcription using the Qiagen RT First Strand Kit and Bio-Rad T100 thermal cycler. qPCR was performed using SsoAdvanced SYBR Green Supermix from Bio-Rad. The primers for GLUT1, GLUT3 and GAPDH were obtained from SABiosciences. The data was analyzed by the ΔΔCt method with GAPDH gene as a housekeeping gene, normalized to control.

Confocal imaging of colocalization

H460 were seeded with 5 × 10^5 cells on a 35 mm cover glass bottom dish with 20 mm micro-well cover glass (Corning). Upon incubated with RPMI 1640 medium for 6h, the cells were washed with PBS and fixed by 4% (w/v) solution of paraformaldehyde for 10 min. Then, cells were treated with 0.1% (v/v) Triton X-100 for 15 min and washed three times by PBS. 1% w/v BSA in TBST was used for blocking and incubated with GLUT1 antibody (Abcam, 1:300 in TBST buffer) for 1h at 37 °C. After washed with PBS for three times (10 min every time), cells were treated with Goat Anti-Rabbit IgG (H+L) antibody (Alexa Fluor® 568, Abcam, 1:400 in TBST buffer, excited at 543 nm) for 40 min, following by washing with PBS for three times (10 min every time). Finally, the cells were treated with 13B-NH_2 (1 μM,

excited at 488 nm, due to the large influence from self-fluorescence of cells at 405nm) for 5 min and washed with PBS for three times. All images were captured by Zeiss LSM780 Inverted Confocal microscope (×60 objective, Zeiss, Germany).

Imaging of 2-NBDG uptake

H460 cells were seeded in a 24-well plate at a density of 1 × 10^5 cells per well for 12h. Then, the cells were washed with PBS and treated with or without 10 μM **TH-G313B** for 0.5 h at 37 °C. Upon 2-NBDG (50 μM, excited at 488 nm) were added for 15 minutes, the cells were washed with PBS three times and imaged on a Nikon Spinning Disc Confocal microscope with ×10 objective.

Apoptosis assay

H460 cells were seeded in a 24-well plate at a density of 1 × 10^5 cells per well and treated respectively with RPMI medium (11 mM glucose, 2% (v/v) FBS, 100 μg/mL streptomycin and 100 U/mL penicillin), low-glucose RPMI medium (2 mM glucose, 2% (v/v) FBS, 100 μg/mL streptomycin and 100 U/mL penicillin) and RPMI medium containing **TH-G313B** (10 μM **TH-G313B**, 11 mM glucose, 2% (v/v) FBS, 100 μg/mL streptomycin and 100 U/mL penicillin) for 24h at 37 °C and 5% CO_2. Then, cells were washed with PBS and collected in centrifugal tube. The cells were resuspend with Binding Buffer (100 μL), added with Annexin V-FITC (2.5 μL) and PI (2.5 μL), followed by incubation in darkness at 37 °C for 30 min. This assay was carried out on LSRFortessa flow cytometer (BD, US).

WST-1 cytotoxicity assay

In a 96-well plate, 10,000 cells in 100 μL media (DMEM medium, supplemented with 10% FBS, 100 μg/mL streptomycin and 100 U/mL penicillin) per well. Eight wells were left empty for blank controls. The plates were incubated (37 ºC, 5% CO_2) overnight to allow the cells to attach to the wells. WST-1 proliferation reagent was added to the cells (10 μL per well) and continued to incubate for 1-2 h at 37 °C. When a clear difference could be seen by naked eye, results were read by spectrophotometer at 490 nm. Cytotoxicity (%) was determined using the following formula: Cytotoxicity (%) = (1 – [Compounds (OD_{490}) – Background (OD_{490})]/[Control (OD_{490}) –Background (OD_{490})]) × 100.

Metabolite extraction and metabolomics

H460 cell lines were plated at 1 × 10^6 cells per well in 6-well plate and incubated with or without **TH-G313B** (10 μM) for 24 h before collection. Two hours before collection, cells were incubated with fresh media. At the time of collection, cells were washed with ice-cold saline, lysed with 80% methanol in water and quickly scraped into an Eppendorf tube followed by three freeze–thaw cycles in liquid nitrogen. The insoluble material was pelleted in a cooled centrifuge (4 °C) and the supernatant was transferred to a new tube and evaporated to dryness using a SpeedVac concentrator (Thermo Savant). So far, we have mainly obtained hydrophilic metabolites of cells.
Dried metabolites were reconstituted in 50 μL of 0.03% formic acid, vortexed, centrifuged at 15,000g for 15 min at 4 °C and the supernatant was analysed using liquid chromatography–tandem mass spectrometry (LC–MS/MS). An Ultra Performance Liquid

Chromatograph (UPLC) system (Waters, ACQUITY UPLC I-Class) was used for liquid chromatography, with an ACQUITY UPLC HSS-T3 UPLC column (150 × 2.1 mm, 1.8 μm, Waters) and the following gradient: 0–3 min 99% mobile phase A; 3–15 min 99–1% A; 15–17 min 1% A; 17–17.1 min 1–99% A; 17.1–20 min 99% A. Mobile Phase A was 0.03% formic acid in water. Mobile Phase B was 0.03% formic acid in acetonitrile. The flow rate was 0.25 ml·min^{-1}, the column was at 35 °C and the samples in the autosampler were at 4 °C. The injection volume was 10 μL. Mass spectrometry was performed with a triple quadrupole mass spectrometer (Waters Xevo TQ-XS) in multiple reaction monitoring (MRM) mode. A total of 262 metabolites were monitored with 165 ion transitions in positive mode and 97 ion transitions in negative mode. It should be noted that this method targets hydrophilic metabolites, so hydrophobic metabolites would not be found by this way. Chromatogram review and peak area integration were performed using Skyline 4.2.0. 19037 (University of Washington). The peak area for each detected metabolite was normalized against the total ion count of that sample. Normalized peak areas were used as variables for multivariate and univariate statistical data analyses. Hierarchical clustering was performed using Morpheus (https://software.broadinstitute.org/morpheus). Principle component analysis, partial least squares discriminant analysis and variable importance in projection analysis were done using SIMCA-P 14.1 (Umetrics). Pathway analysis was performed using Metaboanalyst 4.0 (Xia, 2018)(Chong et al., 2018).

Animal experiments

The *in vivo* xenograft experiments were approved by the Tsinghua University Animal Welfare and Ethical Review Body (approval number: 18-YH1) and accorded with the relevant regulatory standards, especially the 3R system: replacement, reduction, refinement. Male NU nude mice of 6 weeks' age were injected subcutaneously with H460 cells (10^6 cells in 100 μl Matrigel) in the right flank. After the sixth day of tumorigenesis, the mice were divided into vehicle group (n=6) and **TH-G313B** group (n=6), injected daily without/with **TH-G313B** (10mg/kg, 2% Tween80 and 10%DMSO in saline) in situ subcutaneously. Meanwhile, mice were weighed daily and tumor size were measured daily by calipers. The tumor volume was calculated as: $V = W \times L \times (H/2)$. It was not permitted to maintain the mouse when the size of tumor exceeds 20 mm in any direction, and we will euthanize the mouse according to the rules of the Institutional Animal Care and Use Committee. For the last three mice of vehicle group and **TH-G313B** group, the major organs (liver, kidney and spleen) were obtained and fixed by formalin. In addition, we also fixed the organs of xenograft mice without vehicle-treatment and the mice without injection of cancer cells (healthy group). Then, these organs were embedded with paraffin, sectioned and stained by hematoxylin and eosin (H&E) to be observed through Axio Scan. Z1 microscope (Zeiss, Germany).

3. Supplemental References

Bateman, A., Martin, M.J., Orchard, S., Magrane, M., Alpi, E., Bely, B., Bingley, M., Britto, R., Bursteinas, B., Busiello, G., *et al.* (2019). UniProt: a worldwide hub of protein knowledge. Nucleic Acids Res 47, D506-D515.

Chojnacki, S., Cowley, A., Lee, J., Foix, A., and Lopez, R. (2017). Programmatic access to

bioinformatics tools from EMBL-EBI update: 2017. Nucleic Acids Res 45, W550-W553.
Chong, J., Soufan, O., Li, C., Caraus, I., Li, S.Z., Bourque, G., Wishart, D.S., and Xia, J.G. (2018). MetaboAnalyst 4.0: towards more transparent and integrative metabolomics analysis. Nucleic Acids Res 46, W486-W494.
Deng, D., Sun, P., Yan, C., Ke, M., Jiang, X., Xiong, L., Ren, W., Hirata, K., Yamamoto, M., Fan, S.*, et al.* (2015). Molecular basis of ligand recognition and transport by glucose transporters. Nature 526, 391-396.
Deng, D., Xu, C., Sun, P., Wu, J., Yan, C., Hu, M., and Yan, N. (2014). Crystal structure of the human glucose transporter GLUT1. Nature 510, 121-125.
Frisch MJT GW, S.H., Scuseria GE, Robb MA, Cheeseman JR, Scalmani G, Barone V, Mennucci B, Petersson GA, Nakatsuji H, Caricato M, Li X, Hratchian HP, Izmaylov AF, Bloino J, Zheng G, Sonnenberg JL, Hada M, Ehara M, Toyota K, Fukuda, R, Hasegawa J, Ishida M, Nakajima T, Honda Y, Kitao O, Nakai H, Vreven T, Montgomery JA, Jr JEP, Ogliaro F, Bearpark M, Heyd JJ, Brothers E, Kudin KN, Staroverov VN, Keith T, Kobayashi R, Normand J, Raghavachari K, Rendell A, Burant JC, Iyengar SS, Tomasi J, Cossi M, Rega N, Millam JM, Klene M, Knox JE, Cross JB, Bakken V, Adamo C, Jaramillo J, Gomperts R, Stratmann RE, Yazyev O, Austin AJ, Cammi R, Pomelli C, Ochterski JW, Martin RL, Morokuma K, Zakrzewski VG, Voth GA, Salvador P, Dannenberg JJ, Dapprich S, Daniels AD, Farkas O, Foresman JB, Ortiz JV, Cioslowski J, Fox DJ. Gaussian 09 (Gaussian, Inc.).
Goto, H., and Osawa, E. (1993). An efficient algorithm for searching low-energy conformers of cyclic and acyclic molecule. J Chem Soc-Perkin Trans 2, 187-198.
Halgren, T.A., Murphy, R.B., Friesner, R.A., Beard, H.S., Frye, L.L., Pollard, W.T., and Banks, J.L. (2004). Glide: A new approach for rapid, accurate docking and scoring. 2. Enrichment factors in database screening. J Med Chem 47, 1750-1759.
Laskowski, R.A., Macarthur, M.W., Moss, D.S., and Thornton, J.M. (1993). PROCHECK: a program to check the stereochemical quality of protein structures. J Appl Crystallogr 26, 283-291.
Sali, A., and Blundell, T.L. (1993). Comparative protein modelling by satisfaction of spatial restraints. J Mol Biol 234, 779-815.
Schrödinger_Release (2018-1). Schrödinger. LLC, New York, NY, 2018.
Ung, P.M.-U., Song, W., Cheng, L., Zhao, X., Hu, H., Chen, L., and Schlessinger, A.J.A.c.b. (2016). Inhibitor discovery for the human GLUT1 from homology modeling and virtual screening. 11, 1908-1916.

4. Synthesis and Experimental data

Most of the molecules are synthesized by **Method A**, and other special synthetic routes will be described separately with the molecule.

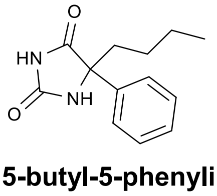

5-butyl-5-phenylimidazolidine-2,4-dione: White solid; ^{1}H NMR (400 MHz, DMSO) δ 10.74 (s, 1H), 8.64 (s, 1H), 7.51 (d, *J* = 7.2 Hz, 2H), 7.40 (t, *J* = 7.5 Hz, 2H), 7.32 (t, *J* = 7.2 Hz, 1H), 2.08 – 1.83 (m, 2H), 1.37 – 1.12 (m, 4H), 0.85 (t, *J* = 7.2 Hz, 3H); ^{13}C NMR (101 MHz, DMSO) δ

176.77, 156.98, 139.76, 128.87, 128.16, 125.78, 67.97, 38.39, 25.97, 22.43, 14.28.

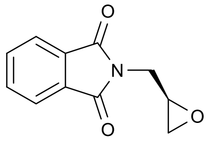

(R)-2-(oxiran-2-ylmethyl)isoindoline-1,3-dione: White solid; ^{1}H NMR (400 MHz, $CDCl_3$) δ 7.93 – 7.84 (m, 2H), 7.80 – 7.72 (m, 2H), 3.98 (dd, *J* = 14.4, 5.0 Hz, 1H), 3.83 (dd, *J* = 14.4, 5.0 Hz, 1H), 3.26 (tdd, *J* = 5.0, 3.9, 2.6 Hz, 1H), 2.86 – 2.78 (m, 1H), 2.71 (dd, *J* = 4.8, 2.5 Hz, 1H); ^{13}C NMR (101 MHz, $CDCl_3$) δ 167.99, 134.15, 131.97, 123.46, 49.08, 46.14, 39.68.

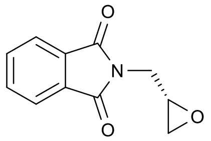

(S)-2-(oxiran-2-ylmethyl)isoindoline-1,3-dione: White solid; ^{1}H NMR (400 MHz, $CDCl_3$) δ 7.94 – 7.84 (m, 2H), 7.80 – 7.71 (m, 2H), 3.98 (dd, *J* = 14.4, 5.0 Hz, 1H), 3.83 (dd, *J* = 14.4, 5.0 Hz, 1H), 3.26 (tdd, *J* = 5.0, 3.9, 2.6 Hz, 1H), 2.85 – 2.81 (m, 1H), 2.71 (dd, *J* = 4.8, 2.5 Hz, 1H); ^{13}C NMR (101 MHz, $CDCl_3$) δ 168.08, 168.00, 134.14, 131.97, 123.46, 49.08, 46.14, 39.68.

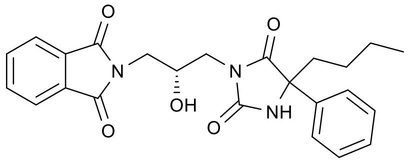

2-((2R)-3-(4-butyl-2,5-dioxo-4-phenylimidazolidin-1-yl)-2-hydroxypropyl)isoindoline-1,3-dione: White solid; ^{1}H NMR (400 MHz, $CDCl_3$) δ 7.86 (m, *J* = 7.3, 3.7 Hz, 2H), 7.80 – 7.70 (m, 2H), 7.53 (d, *J* = 7.7 Hz, 2H), 7.39 (dt, *J* = 24.1, 7.3 Hz, 3H), 5.90 (d, *J* = 3.0 Hz, 1H), 4.23 (m, 1H), 3.84 – 3.64 (m, 4H), 3.37 (dd, *J* = 17.8, 5.5 Hz, 1H), 2.29 – 2.09 (m, 2H), 1.40 – 1.21 (m, 4H), 0.91 (t, *J* = 7.0 Hz, 3H); ^{13}C NMR (101 MHz, $CDCl_3$) δ 175.24, 168.54, 157.35, 137.56, 134.11, 131.93, 128.91, 128.45, 125.33, 123.46, 68.33, 67.61, 43.11, 41.91, 38.34, 25.70, 22.47, 13.77. HRMS (ESI): calcd for: C24H25N3O5 $[M+H]^+$ = 436.1872, obsd $[M+H]^+$ = 436.1868.

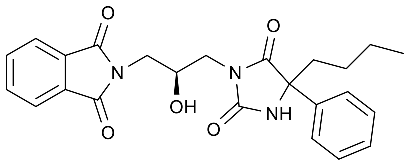

2-((2S)-3-(4-butyl-2,5-dioxo-4-phenylimidazolidin-1-yl)-2-hydroxypropyl)isoindoline-1,3-dione: White solid; ^{1}H NMR (400 MHz, $CDCl_3$) δ 7.86 (m, *J* = 7.0, 3.5 Hz, 2H), 7.80 – 7.68 (m, 2H), 7.54 (d, *J* = 7.7 Hz, 2H), 7.38 (dt, *J* = 24.5, 7.2 Hz, 3H), 6.19 (s, 1H), 4.22 (m, 1H), 3.92 – 3.61 (m, 4H), 3.41 (dd, *J* = 16.7, 5.3 Hz, 1H), 2.30 – 2.06 (m, 2H), 1.42 – 1.21 (m, 4H), 0.97 – 0.82 (t, 3H); ^{13}C NMR (101 MHz, $CDCl_3$) δ 175.17, 168.56, 157.13, 137.50, 134.14, 131.92, 128.94, 128.50, 125.31, 123.48, 68.37, 67.50, 43.06, 41.90, 38.24, 25.70, 22.48, 13.79; HRMS (ESI): calcd for: C24H25N3O5 $[M+H]^+$ = 436.1872, obsd $[M+H]^+$ = 436.1877.

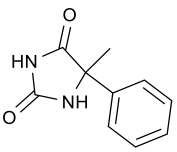

5-methyl-5-phenylimidazolidine-2,4-dione: White solid; ^{1}H NMR (400 MHz, DMSO) δ 10.75 (s, 1H), 8.60 (s, 1H), 7.50 – 7.45 (m, 2H), 7.43 – 7.37 (m, 2H), 7.36 – 7.30 (m, 1H), 1.66 (s, 3H); ^{13}C NMR (101 MHz, DMSO) δ 177.41, 156.68, 140.40, 128.92, 128.26, 125.77, 64.36, 25.42.

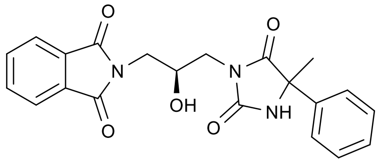

2-((2S)-2-hydroxy-3-(4-methyl-2,5-dioxo-4-phenylimidazolidin-1-yl)propyl)isoindoline-1,3-dione: White solid; ^{1}H NMR (400 MHz, $CDCl_3$) δ 7.91 – 7.80 (m, 2H), 7.77 – 7.70 (m, 2H), 7.54 – 7.49 (m, 2H), 7.43 – 7.32 (m, 3H), 6.24 (s, 1H), 4.29 – 4.20 (m, 1H), 3.85 – 3.41 (m, 5H), 1.89 (s, *J* = 10.1 Hz, 3H); ^{13}C NMR (101 MHz, $CDCl_3$) δ 175.63, 168.58, 156.89, 138.18, 134.14, 131.91, 129.00, 128.64, 125.29, 123.47, 68.22, 63.93, 42.97, 41.83, 25.22; HRMS (ESI): calcd for: C21H19N3O5 $[M+H]^+$ = 394.1403, obsd $[M+H]^+$ = 394.1396.

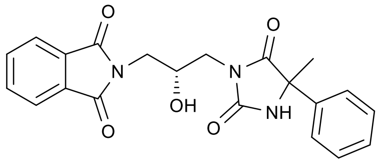

2-((2R)-2-hydroxy-3-(4-methyl-2,5-dioxo-4-phenylimidazolidin-1-yl)propyl)isoindoline-1,3-dione: White solid; ^{1}H NMR (400 MHz, $CDCl_3$) δ 7.94 – 7.81 (m, 2H), 7.81 – 7.68 (m, 2H), 7.57 – 7.48 (m, 2H), 7.46 – 7.33 (m, 3H), 6.00 (s, 1H), 4.25 (m, *J* = 11.9, 5.9 Hz, 1H), 4.00 – 3.52 (m, 5H), 1.90 (s, 3H); ^{13}C NMR (101 MHz, $CDCl_3$) δ 175.60, 168.60, 156.79, 138.15, 134.15, 131.91, 129.01, 128.67, 125.28, 123.48, 68.36, 63.91, 42.97, 41.83, 25.18; HRMS (ESI): calcd for: C21H19N3O5 $[M+H]^+$ = 394.1403, obsd $[M+H]^+$ = 394.1396.

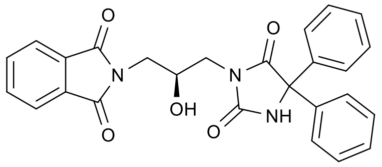

(S)-2-(3-(2,5-dioxo-4,4-diphenylimidazolidin-1-yl)-2-hydroxypropyl)isoindoline-1,3-dione: White solid; ^{1}H NMR (400 MHz, $CDCl_3$) δ 7.87 (m, *J* = 7.0, 3.5 Hz, 2H), 7.79 – 7.69 (m, 2H), 7.40 (m, *J* = 2.4 Hz, 10H), 6.30 (s, 1H), 4.29 (m, 1H), 3.86 – 3.71 (m, 4H), 3.32 (d, *J* = 4.6 Hz, 1H); ^{13}C NMR (101 MHz, $CDCl_3$) δ 173.86, 168.59, 156.47, 138.67, 134.15, 131.92, 128.91, 128.76, 127.01, 123.50, 70.54, 68.38, 43.25, 41.99; HRMS (ESI): calcd for: C26H21N3O5 $[M+H]^+$ = 456.1559, obsd $[M+H]^+$ = 456.1551.

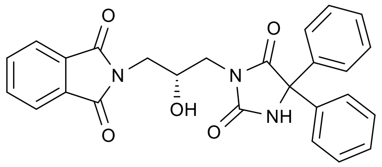

(R)-2-(3-(2,5-dioxo-4,4-diphenylimidazolidin-1-yl)-2-hydroxypropyl)isoindoline-1,3-dione: White solid; ^{1}H NMR (400 MHz, $CDCl_3$) δ 7.90 – 7.83 (m, 2H), 7.78 – 7.71 (m, 2H), 7.55 – 7.31 (m, 10H), 6.32 (s, 1H), 4.34 – 4.26 (m, 1H), 3.88 – 3.71 (m, 4H), 3.31 (d, *J* = 6.2 Hz, 1H); ^{13}C NMR (101 MHz, $CDCl_3$) δ 173.86, 168.58, 156.48, 138.80, 134.15, 131.92, 128.89, 128.75, 127.01, 123.50, 70.54, 68.38, 43.25, 41.99; HRMS (ESI): calcd for: C26H21N3O5 $[M+H]^+$ = 456.1559, obsd $[M+H]^+$ = 456.1552.

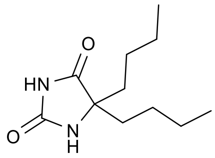

5,5-dibutylimidazolidine-2,4-dione: White solid; ^{1}H NMR (400 MHz, DMSO) δ 10.61 (s, 1H), 7.82 (s, 1H), 1.64 – 1.45 (m, 4H), 1.30 – 0.99 (m, 8H), 0.84 (t, *J* = 7.1 Hz, 6H); ^{13}C NMR (101 MHz, DMSO) δ 178.57, 157.35, 66.07, 36.65, 25.49, 22.58, 14.32.

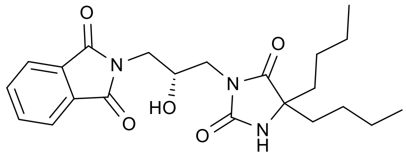

(R)-2-(3-(4,4-dibutyl-2,5-dioxoimidazolidin-1-yl)-2-hydroxypropyl)isoindoline-1,3-dione: White solid; ^{1}H NMR (400 MHz, $CDCl_3$) δ 7.88 (d, *J* = 2.2 Hz, 2H), 7.75 (s, 2H), 5.33 (s, 1H), 4.18 (s, 1H), 3.90 – 3.63 (m, 4H), 3.38 (d, *J* = 32.1 Hz, 1H), 1.81 (m, 2H), 1.64 (m, 2H), 1.24 (m, 8H), 0.88 (t, *J* = 14.7 Hz, 6H); ^{13}C NMR (101 MHz, $CDCl_3$) δ 176.91, 168.59, 157.16, 134.15, 131.95, 123.50, 68.57, 65.95, 42.85, 41.94, 36.84, 25.29, 22.58, 13.82; HRMS (ESI): calcd for: C22H29N3O5 $[M+H]^+$ = 416.2185, obsd $[M+H]^+$ = 416.2192.

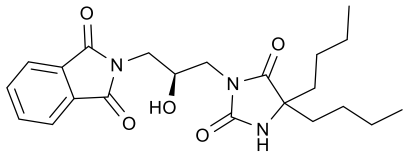

(S)-2-(3-(4,4-dibutyl-2,5-dioxoimidazolidin-1-yl)-2-hydroxypropyl)isoindoline-1,3-dione: White solid; ^{1}H NMR (400 MHz, $CDCl_3$) δ 7.90 – 7.84 (m, 2H), 7.77 – 7.71 (m, 2H), 6.03 (s, 1H), 4.25 – 4.15 (m, 1H), 3.83 – 3.63 (m, 4H), 3.50 (d, *J* = 6.0 Hz, 1H), 1.84 – 1.77 (m, 2H), 1.70 – 1.60 (m, 2H), 1.36 – 1.13 (m, 8H), 0.89 (t, *J* = 7.1, 3.5 Hz, 6H); ^{13}C NMR (101 MHz, $CDCl_3$) δ 177.03, 168.53, 157.58, 134.10, 131.96, 123.44, 68.46, 65.96, 42.85, 41.96, 36.74, 25.29, 22.56, 13.79; HRMS (ESI): calcd for: C22H29N3O5 $[M+H]^+$ = 416.2185, obsd $[M+H]^+$ = 416.2178.

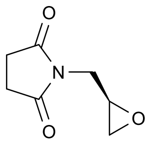

(R)-1-(oxiran-2-ylmethyl)pyrrolidine-2,5-dione: The MeCN solution of pyrrolidine-2,5-dione (1.0 equiv.) and (*S*)-epichlorohydrin (2.0 equiv.) was stirred with NEt_3 (1.5 equiv.) at 80 °C for 6 h. The reaction mixture was diluted and purified by column chromatography to give the final product (yield = 43%). White solid; ^{1}H NMR (400 MHz, $CDCl_3$) δ 3.77 (dd, *J* = 14.0, 5.5 Hz, 1H), 3.66 (dd, *J* = 14.0, 4.8 Hz, 1H), 3.19 (m, *J* = 5.5, 4.8, 3.9, 2.5 Hz, 1H), 2.83 – 2.75 (m, 5H), 2.65 (dd, *J* = 4.8, 2.5 Hz, 1H); ^{13}C NMR (101 MHz, $CDCl_3$) δ 176.84, 48.47, 46.15, 40.63, 28.19.

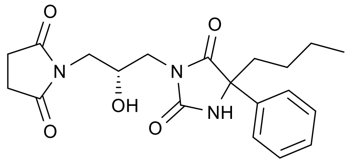

5-butyl-3-((R)-3-(2,5-dioxopyrrolidin-1-yl)-2-hydroxypropyl)-5-phenylimidazolidine-2,4-dione: White solid; ^{1}H NMR (400 MHz, $CDCl_3$) δ 7.52 (d, *J* = 7.7 Hz, 2H), 7.36 (m, *J* = 23.5, 7.2 Hz, 3H), 6.62 (s, 1H), 4.13 (m, *J* = 11.2, 5.9 Hz, 1H), 3.66 – 3.48 (m, 5H), 2.72 (s, 4H), 2.25 – 2.08 (m, 2H), 1.38 – 1.26 (m, 4H), 0.87 (t, *J* = 7.2 Hz, 3H); ^{13}C NMR (101 MHz, $CDCl_3$) δ 177.67, 175.27, 157.27, 137.52, 128.90, 128.45, 125.35, 67.62, 42.90, 42.56, 38.32, 29.70, 28.17, 25.70, 22.47, 13.78; HRMS (ESI): calcd for: C20H25N3O5 $[M+H]^+$ = 388.1872, obsd $[M+H]^+$ = 388.1867.

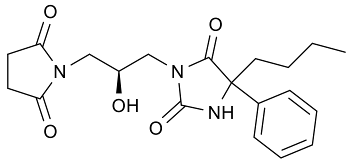

5-butyl-3-((S)-3-(2,5-dioxopyrrolidin-1-yl)-2-hydroxypropyl)-5-phenylimidazolidine-2,4-dione: White solid; ^{1}H NMR (400 MHz, $CDCl_3$) δ 7.54 (d, *J* = 7.4 Hz, 2H), 7.43 – 7.29 (m, 3H), 7.13 (s, 1H), 4.17 – 4.10 (m, 1H), 3.77 – 3.39 (m, 5H), 2.71 (s, 4H), 2.25 – 2.06 (m, 2H), 1.37 – 1.26 (m, 4H), 0.88 (t, *J* = 6.9 Hz, 3H); ^{13}C NMR (101 MHz, $CDCl_3$) δ 177.74, 175.34, 157.43, 137.43, 128.95, 128.52, 125.34, 67.65, 42.88, 42.53, 38.21, 29.73, 28.18, 25.69, 22.49, 13.80; HRMS (ESI): calcd for: C20H25N3O5 $[M+H]^+$ = 388.1872, obsd $[M+H]^+$ = 388.1865.

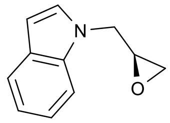

(S)-1-(oxiran-2-ylmethyl)-1H-indole: The mixture of 1H-indole (1.0 equiv.), KOH (4.0 equiv.) and $(C_4H_9)_4NI$ (0.1 equiv.) were stirred in (*R*)-epichlorohydrin (10.0 equiv.) at r.t. for 12 h. The reaction mixture was purified by column chromatography to give the final product (yield = 69%). White solid; ^{1}H NMR (400 MHz, $CDCl_3$) δ 7.70 – 7.64 (m, 1H), 7.42 (m, 1H), 7.28 – 7.22 (m, 1H), 7.19 – 7.12 (m, 2H), 6.56 (dd, *J* = 3.2, 0.8 Hz, 1H), 4.46 (dd, *J* = 15.3, 3.2 Hz, 1H), 4.23 (dd, *J* = 15.3, 5.3 Hz, 1H), 3.32 (dddd, *J* = 5.4, 3.9, 3.2, 2.6 Hz, 1H), 2.84 (dd, *J* = 4.7, 4.0 Hz, 1H), 2.49 (dd, *J* = 4.8, 2.6 Hz, 1H); ^{13}C NMR (101 MHz, $CDCl_3$) δ 136.37, 128.59, 128.25, 121.81, 121.05, 119.66, 109.27, 101.95, 50.92, 47.80, 45.27.

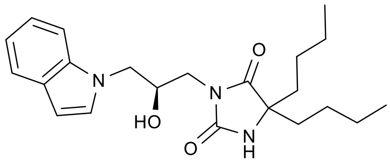

(S)-5,5-dibutyl-3-(2-hydroxy-3-(1H-indol-1-yl)propyl)imidazolidine-2,4-dione: White solid; ^{1}H NMR (400 MHz, $CDCl_3$) δ 7.63 (d, *J* = 7.9 Hz, 1H), 7.38 (d, *J* = 8.2 Hz, 1H), 7.21 (dd, *J* = 9.2, 5.3 Hz, 2H), 7.11 (t, *J* = 7.4 Hz, 1H), 6.52 (d, *J* = 2.9 Hz, 1H), 5.50 (s, 1H), 4.27 – 4.21 (m, 2H), 4.14 (m, 1H), 3.74 – 3.56 (m, 2H), 3.04 (d, *J* = 4.2 Hz, 1H), 1.78 (dd, *J* = 16.2, 9.1 Hz, 2H), 1.62 – 1.56 (m, 2H), 1.29 (m, 6H), 1.10 (dd, *J* = 7.9, 3.5 Hz, 2H), 0.87 (dt, *J* = 6.9, 3.4 Hz, 6H); ^{13}C NMR (101 MHz, $CDCl_3$) δ 177.14, 157.76, 136.36, 128.73, 128.66, 121.75, 121.05, 119.61, 109.43, 101.83, 69.68, 66.00, 50.22, 42.51, 36.78, 25.34, 22.54, 13.85; HRMS (ESI): calcd for: C22H31N3O3 $[M+H]^+$ = 386.2444, obsd $[M+H]^+$ = 386.2439.

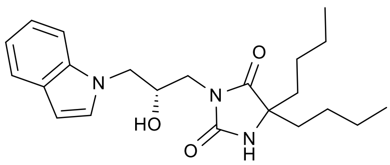

(R)-5,5-dibutyl-3-(2-hydroxy-3-(1H-indol-1-yl)propyl)imidazolidine-2,4-dione: White solid; ^{1}H NMR (400 MHz, $CDCl_3$) δ 7.63 (d, *J* = 7.9 Hz, 1H), 7.38 (d, *J* = 8.2 Hz, 1H), 7.21 (dd, *J* = 8.8, 5.3 Hz, 2H), 7.11 (t, *J* = 7.5 Hz, 1H), 6.53 (d, *J* = 3.1 Hz, 1H), 5.39 (s, 1H), 4.24 (dt, *J* = 7.2, 3.9 Hz, 2H), 4.14 (dd, *J* = 15.4, 8.0 Hz, 1H), 3.74 – 3.56 (m, 2H), 3.00 (d, *J* = 4.3 Hz, 1H), 1.84 – 1.73 (m, 2H), 1.62 (m, 2H), 1.29 (m, 6H), 1.14 – 1.06 (m, 2H), 0.87 (td, *J* = 6.9, 1.7 Hz, 6H); ^{13}C NMR (101 MHz, $CDCl_3$) δ 176.93, 157.33, 136.33, 128.68, 128.66, 121.77, 121.07, 119.61, 109.38, 101.91, 69.85, 66.00, 50.22, 42.52, 36.87, 25.33, 22.53, 13.82; HRMS (ESI): calcd for: C22H31N3O3 $[M+H]^+$ = 386.2444, obsd $[M+H]^+$ = 386.2438.

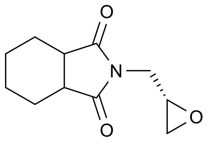

2-(((S)-oxiran-2-yl)methyl)hexahydro-1H-isoindole-1,3(2H)-dione: Following the method used for **(R)-1-(oxiran-2-ylmethyl)pyrrolidine-2,5-dione** to give the final product (yield = 46%). White solid; ^{1}H NMR (400 MHz, $CDCl_3$) δ 3.69 (ddd, *J* = 47.2, 14.0, 5.0 Hz, 2H), 3.16 (tdd, *J* = 5.0, 3.9, 2.6 Hz, 1H), 2.95 – 2.85 (m, 2H), 2.81 – 2.58 (m, 2H), 1.96 – 1.68 (m, 4H), 1.55 – 1.36 (m, 4H); ^{13}C NMR (101 MHz, $CDCl_3$) δ 179.46, 48.60, 46.03, 40.00, 39.84, 23.69, 21.63.

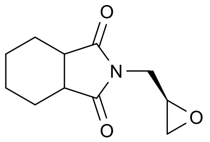

2-(((R)-oxiran-2-yl)methyl)hexahydro-1H-isoindole-1,3(2H)-dione: White solid; ^{1}H NMR (400 MHz, $CDCl_3$) δ 3.77 – 3.60 (m, 2H), 3.16 (ddd, *J* = 8.9, 5.0, 2.6 Hz, 1H), 2.94 – 2.87 (m, 2H), 2.68 (ddd, *J* = 7.4, 6.8, 3.5 Hz, 2H), 1.92 – 1.72 (m, 4H), 1.51 – 1.40 (m, 4H); ^{13}C NMR (101 MHz, $CDCl_3$) δ 179.49, 48.61, 46.01, 39.98, 39.82, 23.94, 21.64.

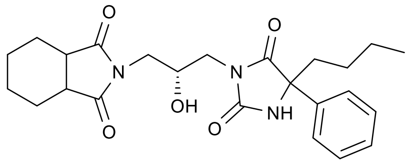

2-((2R)-3-(4-butyl-2,5-dioxo-4-phenylimidazolidin-1-yl)-2-hydroxypropyl)hexahydro-1H-isoindole-1,3(2H)-dione: White solid; ^{1}H NMR (400 MHz, $CDCl_3$) δ 7.54 (d, *J* = 7.4 Hz, 2H), 7.38 (dt, *J* = 24.1, 7.2 Hz, 3H), 6.45 (d, *J* = 3.7 Hz, 1H), 4.16 – 4.10 (m, 1H), 3.80 – 3.36 (m, 5H), 2.90 (m, 2H), 2.18 (m, 2H), 1.83 (m, 4H), 1.49 – 1.28 (m, 8H), 0.90 (t, *J* = 7.1 Hz, 3H); ^{13}C NMR (101 MHz, $CDCl_3$) δ 180.20, 175.16, 157.20, 137.53, 128.91, 128.45, 125.34, 67.91, 43.01, 42.19, 39.76, 38.30, 29.70, 25.69, 23.81, 22.47, 21.60, 13.77; HRMS (ESI): calcd for: C24H31N3O5 $[M+H]^+$ = 442.2342, obsd $[M+H]^+$ = 442.2345.

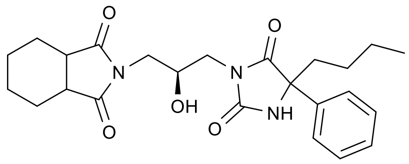

2-((2S)-3-(4-butyl-2,5-dioxo-4-phenylimidazolidin-1-yl)-2-hydroxypropyl)hexahydro-1H-isoindole-1,3(2H)-dione: White solid; ^{1}H NMR (400 MHz, $CDCl_3$) δ 7.60 – 7.49 (m, 2H), 7.38 (dt, *J* = 23.7, 7.3 Hz, 3H), 6.31 (d, *J* = 3.9 Hz, 1H), 4.16 – 4.07 (m, 1H), 3.70 – 3.38 (m, 5H), 2.98 – 2.84 (m, 2H), 2.26 – 2.08 (m, 2H), 1.93 – 1.75 (m, 4H), 1.50 – 1.28 (m, 8H), 0.90 (t, *J* = 7.1 Hz, 3H); ^{13}C NMR (101 MHz, $CDCl_3$) δ 180.19, 175.13, 157.13, 137.52, 128.91, 128.46, 125.34, 67.92, 42.98, 42.20, 39.81, 38.28, 29.69, 25.69, 23.79, 22.47, 21.59, 13.75; HRMS (ESI): calcd for: C24H31N3O5 $[M+H]^+$ = 442.2342, obsd $[M+H]^+$ = 442.2347.

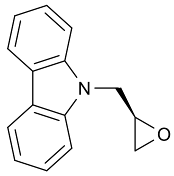

(R)-9-(oxiran-2-ylmethyl)-9H-carbazole: Following the method used for **(S)-1-(oxiran-2-ylmethyl)-1H-indole** to give the final product (yield = 87%). White solid; ^{1}H NMR (400 MHz, $CDCl_3$) δ 8.13 (dt, *J* = 7.8, 0.9 Hz, 2H), 7.54 – 7.48 (m, 4H), 7.31 – 7.27 (m, 2H), 4.66 (dd, *J* = 15.8, 3.4 Hz, 1H), 4.43 (dd, *J* = 15.8, 4.8 Hz, 1H), 3.38 (tdd, *J* = 4.8, 3.6, 2.6 Hz, 1H), 2.84 (dd, *J* = 4.7, 4.0 Hz, 1H), 2.60 (dd, *J* = 4.8, 2.6 Hz, 1H); ^{13}C NMR (101 MHz, $CDCl_3$) δ 140.72, 125.91, 123.03, 120.37, 119.39, 108.78, 50.59, 45.38, 44.63.

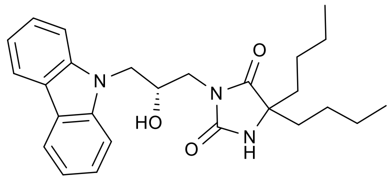

(R)-3-(3-(9H-carbazol-9-yl)-2-hydroxypropyl)-5,5-dibutylimidazolidine-2,4-dione: White solid; ^{1}H NMR (400 MHz, $CDCl_3$) δ 8.11 (d, *J* = 7.6 Hz, 2H), 7.49 (m, *J* = 7.8 Hz, 4H), 7.28 – 7.24 (m, 2H), 5.36 (d, *J* = 9.2 Hz, 1H), 4.49 – 4.36 (m, 3H), 3.79 (ddd, *J* = 21.3, 14.2, 5.1 Hz, 2H), 2.89 (s, 1H), 1.81 (m, 2H), 1.61 (m, 2H), 1.35 – 1.28 (m, 6H), 1.14 (m, 2H), 0.89 (td, *J* =

7.0, 2.5 Hz, 6H); ^{13}C NMR (101 MHz, $CDCl_3$) δ 176.82, 157.24, 140.85, 125.89, 123.12, 120.35, 119.37, 109.04, 69.83, 65.94, 47.74, 42.92, 36.83, 25.33, 22.53, 13.81; HRMS (ESI): calcd for: C26H33N3O3 $[M+H]^+$ = 436.2600, obsd $[M+H]^+$ = 436.2592.

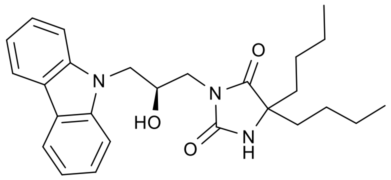

(S)-3-(3-(9H-carbazol-9-yl)-2-hydroxypropyl)-5,5-dibutylimidazolidine-2,4-dione: White solid; ^1H NMR (400 MHz, $CDCl_3$) δ 8.11 (d, *J* = 7.6 Hz, 2H), 7.54 – 7.43 (m, 4H), 7.30 – 7.26 (m, 2H), 5.92 (s, 1H), 4.41 (m, 3H), 3.87 – 3.67 (m, 2H), 3.03 (s, 1H), 1.83 – 1.76 (m, 2H), 1.60 (m, 2H), 1.29 (m, 6H), 1.13 (m, 2H), 0.89 (m, 6H); ^{13}C NMR (101 MHz, $CDCl_3$) δ 176.89, 157.34, 140.83, 125.89, 123.08, 120.36, 119.36, 109.06, 69.79, 65.96, 47.71, 42.89, 36.81, 25.32, 22.54, 13.85; HRMS (ESI): calcd for: C26H33N3O3 $[M+H]^+$ = 436.2600, obsd $[M+H]^+$ = 436.2603.

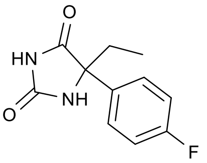

5-ethyl-5-(4-fluorophenyl)imidazolidine-2,4-dione: White solid; ^1H NMR (400 MHz, DMSO) δ 10.65 (s, 1H), 8.70 (s, 1H), 7.53 (ddd, *J* = 8.5, 5.3, 2.6 Hz, 2H), 7.23 (dd, *J* = 12.3, 5.5 Hz, 2H), 2.08 – 1.85 (m, 2H), 0.80 (t, *J* = 7.3 Hz, 3H); ^{13}C NMR (101 MHz, DMSO) δ 176.66, 160.92, 157.03, 135.70, 128.05, 115.78, 68.08, 31.78, 8.44.

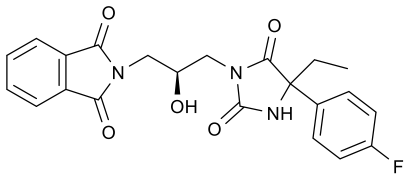

2-((2S)-3-(4-ethyl-4-(4-fluorophenyl)-2,5-dioxoimidazolidin-1-yl)-2-hydroxypropyl)isoindoline-1,3-dione: White solid; ^1H NMR (400 MHz, $CDCl_3$) δ 7.94 – 7.82 (m, 2H), 7.76 (m, 2H), 7.51 (m, 2H), 7.09 (m, 2H), 5.95 (s, 1H), 4.23 (m, 1H), 3.89 – 3.62 (m, 4H), 3.29 (dd, *J* = 18.3, 6.1 Hz, 1H), 2.24 (m, 2H), 1.05 – 0.86 (t, 3H); ^{13}C NMR (101 MHz, $CDCl_3$) δ 175.09, 168.62, 161.48, 157.36, 134.21, 133.23, 131.86, 127.38, 123.50, 115.94, 68.25, 67.56, 42.95, 41.91, 31.89, 8.05; HRMS (ESI): calcd for: C22H20FN3O5 $[M+H]^+$ = 426.1465, obsd $[M+H]^+$ = 426.1463.

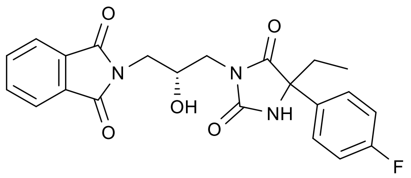

2-((2R)-3-(4-ethyl-4-(4-fluorophenyl)-2,5-dioxoimidazolidin-1-yl)-2-hydroxypropyl)isoindoline-1,3-dione: White solid; ^1H NMR (400 MHz, $CDCl_3$) δ 7.86 (dd, *J* = 5.3, 3.0 Hz, 2H), 7.74 (dd, *J* = 5.4, 3.0 Hz, 2H), 7.53 (m, 2H), 7.10 (m, 2H), 6.31 (d, *J* = 5.1

Hz, 1H), 4.25 – 4.19 (m, 1H), 3.82 – 3.68 (m, 4H), 3.32 (dd, *J* = 16.2, 6.2 Hz, 1H), 2.33 – 2.11 (m, 2H), 0.96 (t, *J* = 7.2 Hz, 3H); ^{13}C NMR (101 MHz, $CDCl_3$) δ 175.28, 168.60, 160.32, 157.42, 134.23, 134.17, 131.86, 127.41, 123.46, 115.85, 68.14, 67.55, 41.89, 40.83, 31.97, 8.05; HRMS (ESI): calcd for: C22H20FN3O5 $[M+H]^+$ = 426.1465, obsd $[M+H]^+$ = 426.1461.

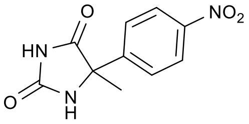

5-methyl-5-(4-nitrophenyl)imidazolidine-2,4-dione: White solid; ^{1}H NMR (400 MHz, DMSO) δ 8.82 (s, 1H), 8.32 – 8.20 (m, 2H), 7.83 – 7.74 (m, 2H), 1.71 (s, 3H). ^{13}C NMR (101 MHz, DMSO) δ 176.47, 156.55, 147.57, 147.42, 127.36, 124.07, 64.47, 25.57.

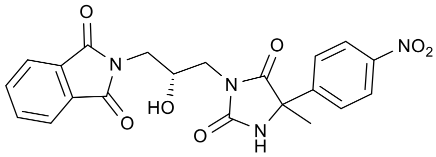

2-((2R)-2-hydroxy-3-(4-methyl-4-(4-nitrophenyl)-2,5-dioxoimidazolidin-1-yl)propyl)isoindoline-1,3-dione: White solid; ^{1}H NMR (400 MHz, Chloroform-*d*) δ 8.28 (d, *J* = 7.4 Hz, 2H), 7.88 – 7.86 (m, 2H), 7.79 – 7.75 (m, 4H), 6.36 (s, 1H), 4.26 (s, 1H), 3.82 – 3.73 (m, 4H), 3.38 (t, *J* = 15.7 Hz, 1H), 1.94 (s, 3H); ^{13}C NMR (101 MHz, $CDCl_3$) δ 174.54, 168.63, 156.73, 147.90, 145.32, 134.26, 131.79, 126.68, 124.01, 123.50, 67.95, 63.77, 41.89, 40.89, 25.81. HRMS (ESI): calcd for: C21H18N4O7 $[M+H]^+$ = 439.1254, obsd $[M+H]^+$ = 439.1244.

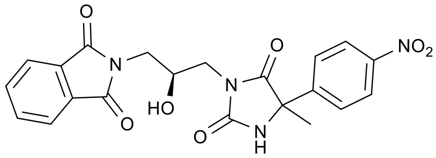

2-((2S)-2-hydroxy-3-(4-methyl-4-(4-nitrophenyl)-2,5-dioxoimidazolidin-1-yl)propyl)isoindoline-1,3-dione: White solid; ^{1}H NMR (400 MHz, $CDCl_3$) δ 8.34 – 8.20 (m, 2H), 7.87 – 7.73 (m, 6H), 6.69 (s, 1H), 4.21 (d, *J* = 26.7 Hz, 1H), 3.81 – 3.67 (m, 4H), 3.49 – 3.34 (m, 1H), 2.00 – 1.89 (s, 3H); ^{13}C NMR (101 MHz, $CDCl_3$) δ 174.76, 168.65, 156.99, 147.78, 145.47, 134.27, 131.76, 126.71, 123.96, 123.48, 67.81, 63.77, 41.86, 40.75, 25.82; HRMS (ESI): calcd for: C21H18N4O7 $[M+H]^+$ = 439.1254, obsd $[M+H]^+$ = 439.1247.

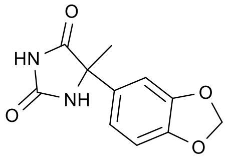

5-(benzo[d][1,3]dioxol-5-yl)-5-methylimidazolidine-2,4-dione: White solid; ^{1}H NMR (400 MHz, DMSO) δ 8.59 (s, 1H), 6.99 (s, 1H), 6.96 – 6.83 (m, 2H), 6.02 (s, 2H), 1.60 (s, 3H); ^{13}C NMR (101 MHz, DMSO) δ 177.46, 156.61, 147.91, 147.27, 134.23, 119.04, 108.44, 106.54, 101.69, 64.17, 25.65.

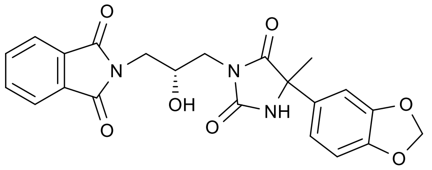

2-((2R)-3-(4-(benzo[d][1,3]dioxol-5-yl)-4-methyl-2,5-dioxoimidazolidin-1-yl)-2-hydroxypropyl)isoindoline-1,3-dione: White solid; ^{1}H NMR (400 MHz, $CDCl_3$) δ 7.84 (d, *J* = 5.7 Hz, 2H), 7.74 (s, 2H), 7.12 – 6.63 (m, 4H), 5.93 (s, 2H), 4.23 (m, 1H), 3.77 (m, 5H), 2.02 – 1.83 (s, 3H); ^{13}C NMR (101 MHz, $CDCl_3$) δ 175.89, 168.59, 156.84, 148.20, 147.79, 134.15, 132.06, 131.87, 123.46, 118.82, 108.36, 106.28, 101.43, 68.07, 63.69, 42.93, 41.81, 25.25; HRMS (ESI): calcd for: C22H19N3O7 $[M+H]^+$ = 438.1301, obsd $[M+H]^+$ = 438.1294.

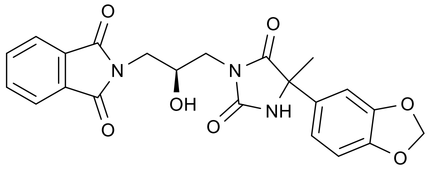

2-((2S)-3-(4-(benzo[d][1,3]dioxol-5-yl)-4-methyl-2,5-dioxoimidazolidin-1-yl)-2-hydroxypropyl)isoindoline-1,3-dione: White solid; ^{1}H NMR (400 MHz, $CDCl_3$) δ 7.87 (dd, *J* = 4.5, 3.0 Hz, 2H), 7.75 (dd, *J* = 4.9, 2.6 Hz, 2H), 7.04 – 6.94 (m, 2H), 6.83 (s, 1H), 5.99 (s, 2H), 5.86 (s, 1H), 4.25 (m, 1H), 3.84 – 3.67 (m, 4H), 3.42 (m, 1H), 1.86 (s, 3H); ^{13}C NMR (101 MHz, $CDCl_3$) δ 175.72, 168.59, 156.74, 148.24, 147.83, 134.13, 132.06, 131.89, 123.45, 118.81, 108.36, 106.26, 101.43, 68.24, 63.67, 42.95, 41.82, 25.23; HRMS (ESI): calcd for: C22H19N3O7 $[M+H]^+$ = 438.1301, obsd $[M+H]^+$ = 438.1294.

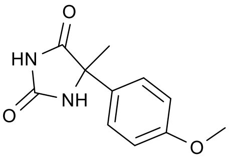

5-(4-methoxyphenyl)-5-methylimidazolidine-2,4-dione: White solid; ^{1}H NMR (400 MHz, DMSO) δ 8.58 (s, 1H), 7.38 – 7.35 (m, 2H), 7.00 – 6.88 (m, 2H), 3.74 (s, 3H), 1.61 (s, 3H); ^{13}C NMR (101 MHz, DMSO) δ 177.76, 159.28, 156.71, 132.28, 127.02, 114.26, 63.99, 55.62, 25.31.

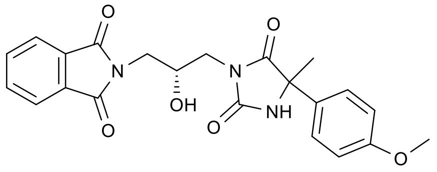

2-((2R)-2-hydroxy-3-(4-(4-methoxyphenyl)-4-methyl-2,5-dioxoimidazolidin-1-yl)propyl)isoindoline-1,3-dione: White solid; ^{1}H NMR (400 MHz, $CDCl_3$) δ 7.96 – 7.84 (m, 2H), 7.75 (dd, *J* = 5.3, 2.3 Hz, 2H), 7.42 (dd, *J* = 6.4, 2.5 Hz, 2H), 6.94 (d, *J* = 8.8 Hz, 2H), 5.77 (s, 1H), 4.26 (s, 1H), 3.97 – 3.61 (m, 7H), 3.49 – 3.40 (m, 1H), 1.89 (s, 3H); ^{13}C NMR (101 MHz, $CDCl_3$) δ 175.87, 168.59, 159.81, 156.75, 134.15, 131.91, 130.10, 126.60, 123.48, 114.36, 68.28, 63.57, 55.36, 42.95, 41.82, 24.99; HRMS (ESI): calcd for: C22H21N3O6 $[M+H]^+$ = 424.1509, obsd $[M+H]^+$ = 424.1509.

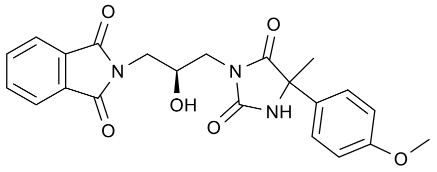

2-((2S)-2-hydroxy-3-(4-(4-methoxyphenyl)-4-methyl-2,5-dioxoimidazolidin-1-yl)propyl)isoindoline-1,3-dione: White solid; ^{1}H NMR (400 MHz, $CDCl_3$) δ 7.87 (m, 2H), 7.75 (m, 2H), 7.42 (d, *J* = 8.0 Hz, 2H), 6.93 (d, *J* = 8.1 Hz, 2H), 6.00 (s, 1H), 4.13 (m, 1H), 3.84 – 3.69 (m, 7H), 3.48 (m, 1H), 1.88 (s, 3H); ^{13}C NMR (101 MHz, $CDCl_3$) δ 176.19, 168.62, 159.77, 156.86, 134.18, 131.89, 130.18, 126.62, 123.49, 114.34, 68.38, 63.56, 55.37, 42.94, 41.81, 24.97. HRMS (ESI): calcd for: C22H21N3O6 $[M+H]^+$ = 424.1509, obsd $[M+H]^+$ = 424.1507.

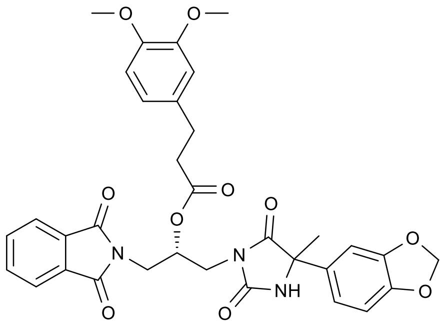

(2S)-1-(4-(benzo[d][1,3]dioxol-5-yl)-4-methyl-2,5-dioxoimidazolidin-1-yl)-3-(1,3-dioxoisoindolin-2-yl)propan-2-yl 3-(3,4-dimethoxyphenyl)propanoate: White solid; ^{1}H NMR (400 MHz, $CDCl_3$) δ 7.72 (d, *J* = 2.8 Hz, 2H), 7.62 (s, 2H), 6.91 – 6.82 (m, 2H), 6.61 (m, 5H), 5.82 – 5.66 (m, 2H), 5.31 (s, 1H), 3.83 – 3.66 (m, 10H), 2.65 – 2.49 (m, 2H), 2.31 (m, 2H), 1.63 (s, 3H); ^{13}C NMR (101 MHz, $CDCl_3$) δ 175.26, 172.25, 167.93, 156.15, 148.83, 148.18, 147.73, 147.38, 134.09, 133.14, 132.35, 131.83, 123.38, 120.01, 118.81, 111.74, 111.34, 108.28, 106.23, 101.36, 69.34, 63.49, 55.89, 40.02, 39.10, 35.68, 29.96, 24.91; HRMS (ESI): calcd for: C33H31N3O10 $[M+Na]^+$ = 630.2088, obsd $[M+H]^+$ = 630.2067.

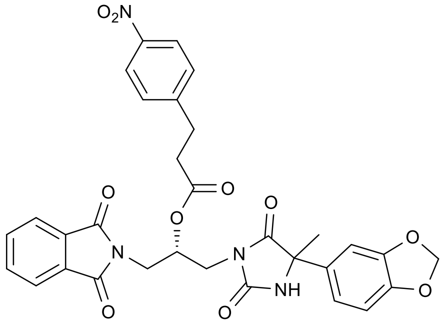

(2S)-1-(4-(benzo[d][1,3]dioxol-5-yl)-4-methyl-2,5-dioxoimidazolidin-1-yl)-3-(1,3-dioxoisoindolin-2-yl)propan-2-yl 3-(4-nitrophenyl)propanoate: White solid; ^{1}H NMR (400 MHz, $CDCl_3$) δ 8.04 (d, *J* = 8.0 Hz, 2H), 7.82 (m, 2H), 7.77 – 7.71 (m, 2H), 7.25 (m, 2H), 7.00 – 6.93 (m, 2H), 6.82 – 6.75 (m, 1H), 6.42 (d, *J* = 3.2 Hz, 1H), 5.91 (m, 2H), 5.39 (m, 1H), 3.95 – 3.74 (m, 4H), 2.93 – 2.77 (m, 2H), 2.58 – 2.36 (m, 2H), 1.79 (s, 3H); ^{13}C NMR (101 MHz, $CDCl_3$) δ 175.24, 171.57, 167.91, 156.09, 148.25, 148.20, 147.84, 146.44, 134.24, 132.09, 131.69, 129.06, 123.59, 123.45, 118.84, 108.39, 106.20, 101.47, 69.87, 63.53, 40.08, 39.04, 34.52, 29.98, 24.92; HRMS (ESI): calcd for: C31H26N4O10 $[M+Na]^+$ = 637.1547, obsd $[M+Na]^+$ = 637.1521.

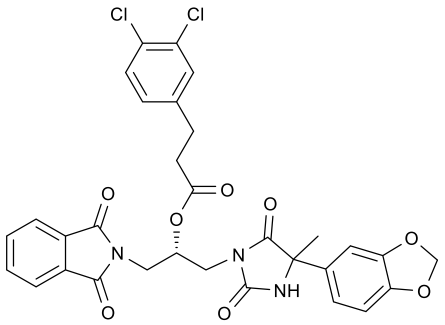

(2S)-1-(4-(benzo[d][1,3]dioxol-5-yl)-4-methyl-2,5-dioxoimidazolidin-1-yl)-3-(1,3-dioxoisoindolin-2-yl)propan-2-yl 3-(3,4-dichlorophenyl)propanoate: White solid; ^{1}H NMR (400 MHz, $CDCl_3$) δ 7.84 (m, 2H), 7.78 – 7.73 (m, 2H), 7.28 – 7.18 (m, 2H), 6.99 – 6.92 (m, 3H), 6.80 (m, 1H), 6.33 – 6.25 (m, 1H), 5.91 (m, 2H), 5.43 – 5.36 (m, 1H), 3.97 – 3.74 (m, 4H), 2.69 (m, 2H), 2.50 – 2.32 (m, 2H), 1.78 (s, 3H); ^{13}C NMR (101 MHz, $CDCl_3$) δ 175.19, 171.70, 167.95, 156.04, 148.23, 147.85, 140.68, 134.22, 132.18, 132.08, 131.75, 130.24, 130.05, 127.68, 123.46, 118.82, 118.67, 108.39, 106.20, 101.46, 69.75, 63.51, 40.14, 39.04, 34.89, 29.33, 24.87; HRMS (ESI): calcd for: C31H25Cl2N3O8 $[M+H]^+$ = 638.1097, obsd $[M+H]^+$ = 638.1095.

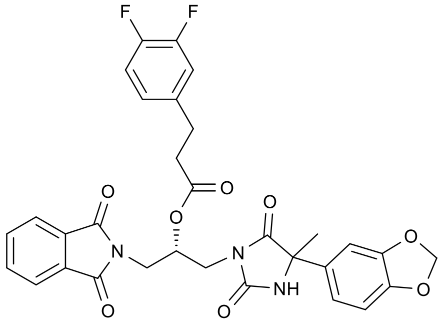

(2S)-1-(4-(benzo[d][1,3]dioxol-5-yl)-4-methyl-2,5-dioxoimidazolidin-1-yl)-3-(1,3-dioxoisoindolin-2-yl)propan-2-yl 3-(3,4-difluorophenyl)propanoate: White solid; ^{1}H NMR (400 MHz, $CDCl_3$) δ 7.89 – 7.66 (m, 4H), 7.30 (m, 1H), 7.13 (dt, *J* = 27.9, 7.0 Hz, 2H), 6.90 (m, 2H), 6.76 (t, *J* = 6.5 Hz, 1H), 5.92 – 5.73 (m, 2H), 5.55 – 5.30 (m, 2H), 4.03 – 3.69 (m, 4H), 2.91 (dt, *J* = 25.1, 8.0 Hz, 2H), 2.65 – 2.47 (m, 2H), 1.67 (m, 3H); ^{13}C NMR (101 MHz, $CDCl_3$) δ 174.98, 171.78, 167.96, 155.92, 148.24, 147.87, 137.36, 134.19, 132.18, 131.82, 131.76, 124.00, 123.45, 118.81, 118.67, 117.10, 116.82, 108.37, 106.25, 101.44, 69.60, 63.49, 41.16, 38.83, 35.31, 29.26, 25.14; HRMS (ESI): calcd for: C31H25F2N3O8 $[M-H]^-$ = 604.1531, obsd $[M-H]^-$ = 604.1528.

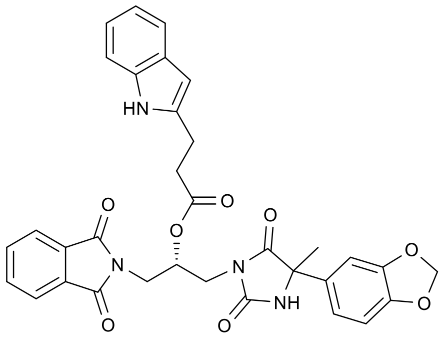

(2S)-1-(4-(benzo[d][1,3]dioxol-5-yl)-4-methyl-2,5-dioxoimidazolidin-1-yl)-3-(1,3-dioxoisoindolin-2-yl)propan-2-yl 3-(1H-indol-2-yl)propanoate: White solid; ^{1}H NMR (400 MHz, $CDCl_3$) δ 7.96 (s, 1H), 7.80 (s, 2H), 7.71 (d, *J* = 2.9 Hz, 2H), 7.54 (d, *J* = 7.4 Hz, 1H), 7.30 (d, *J* = 10.2 Hz, 1H), 7.13 (dt, *J* = 14.6, 7.1 Hz, 2H), 7.07 – 6.84 (m, 3H), 6.82 – 6.65 (m, 1H), 5.84 (m, 2H), 5.45 (s, 1H), 4.12 – 3.67 (m, 4H), 3.04 – 2.82 (m, 2H), 2.55 (m, 2H), 1.69 (s, 3H); ^{13}C NMR (101 MHz, $CDCl_3$) δ 175.02, 172.73, 167.98, 155.99, 148.21, 147.81, 136.22, 134.09, 131.80, 127.19, 123.41, 121.94, 121.23, 119.21, 118.85, 118.77, 118.67, 114.94, 111.01, 108.27, 106.18, 101.37, 69.22, 63.48, 40.24, 38.90, 34.41, 25.00, 20.02; HRMS (ESI): calcd for: C33H28N4O8 $[M+H]^+$ = 609.1985, obsd $[M+H]^+$ = 609.1960.

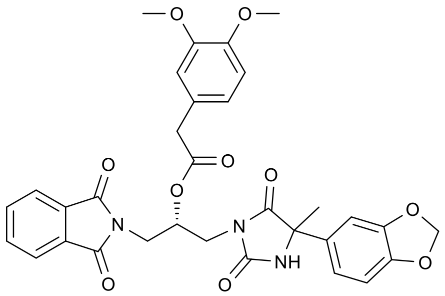

(2S)-1-(4-(benzo[d][1,3]dioxol-5-yl)-4-methyl-2,5-dioxoimidazolidin-1-yl)-3-(1,3-dioxoisoindolin-2-yl)propan-2-yl 2-(3,4-dimethoxyphenyl)acetate: White solid; ^{1}H NMR (400 MHz, $CDCl_3$) δ 7.77 (m, 4H), 6.98 – 6.64 (m, 6H), 5.97 (s, 2H), 5.65 (s, 1H), 5.41 (m, 1H), 3.96 – 3.77 (m, 10H), 3.48 – 3.29 (m, 2H), 1.79 – 1.69 (s, 3H); ^{13}C NMR (101 MHz, $CDCl_3$) δ 175.18, 171.28, 167.81, 156.07, 148.58, 148.22, 147.85, 147.78, 133.99, 132.18, 131.66, 125.99, 123.35, 121.45, 118.82, 112.65, 110.91, 108.35, 106.25, 101.42, 69.39, 63.51, 55.72, 40.53, 39.99, 38.70, 24.89; HRMS (ESI): calcd for: C32H29N3O10 $[M+H]^+$ = 616.1931, obsd $[M+H]^+$ = 616.1935. In addition, for **TH-G313B**, $[a]^{20}_D$ = +10.1 (c = 0.25, $CHCl_3$).

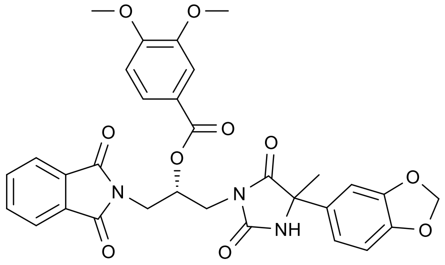

(2S)-1-(4-(benzo[d][1,3]dioxol-5-yl)-4-methyl-2,5-dioxoimidazolidin-1-yl)-3-(1,3-dioxoisoindolin-2-yl)propan-2-yl 3,4-dimethoxybenzoate: White solid; ^{1}H NMR (400 MHz, $CDCl_3$) δ 7.89 – 7.69 (m, 4H), 7.46 (s, 1H), 7.23 (s, 1H), 7.06 – 6.70 (m, 4H), 6.51 (s, 1H), 5.91 (s, 2H), 5.59 (m, 1H), 4.12 – 3.80 (m, 10H), 1.74 (s, 3H); ^{13}C NMR (101 MHz, $CDCl_3$) δ 175.00, 167.97, 165.53, 155.98, 153.13, 148.25, 148.02, 147.54, 134.11, 132.25, 131.92, 123.92, 123.46, 121.83, 118.30, 112.06, 110.12, 108.01, 105.95, 101.42, 69.72, 63.54, 55.95, 39.91, 38.93, 24.80; HRMS (ESI): calcd for: C31H27N3O10 $[M+H]^+$ = 602.1775, obsd $[M+H]^+$ = 602.1782.

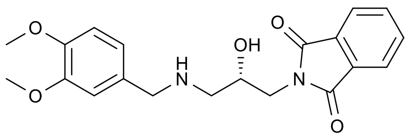

(S)-2-(3-((3,4-dimethoxybenzyl)amino)-2-hydroxypropyl)isoindoline-1,3-dione: The mix of **(S)-2-(oxiran-2-ylmethyl)isoindoline-1,3-dione** (1.0 equiv.) and (3,4-dimethoxyphenyl)methanamine (1.0 equiv.) was stirred with Cs_2CO_3 (4.0 equiv.) in dry DMF at r.t. for 20 h. The reaction mixture was purified by column chromatography to give the final product (yield = 34%).White solid; ^{1}H NMR (400 MHz, $CDCl_3$) δ 7.92 (d, *J* = 5.5 Hz, 2H), 7.64 (dd, *J* = 5.3, 2.5 Hz, 2H), 6.96 (dd, *J* = 15.0, 8.6 Hz, 2H), 6.82 – 6.75 (m, 1H), 4.97 – 4.81 (m, 2H), 4.09 – 3.74 (m, 12H); ^{13}C NMR (101 MHz, $CDCl_3$) δ 167.42, 149.00, 148.43, 133.05, 132.01, 125.63, 123.82, 120.71, 111.85, 111.10, 71.35, 64.73, 55.91, 52.63, 42.00; HRMS (ESI): calcd for: C20H22N2O5 $[M+H]^+$ = 371.1607, obsd $[M+H]^+$ = 371.1604.

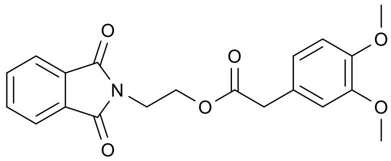

2-(1,3-dioxoisoindolin-2-yl)ethyl 2-(3,4-dimethoxyphenyl)acetate: The mixture of 2-iodoethan-1-ol (1.0 equiv.) and potassium phthalimide (1.5 equiv.) was stirred in DMF at r.t. for 16 h to give 2-(2-hydroxyethyl)isoindoline-1,3-dione. To a stirred solution of the alcohols (1.0 equiv.) and 3,4-Dimethoxyphenyl acetic acid (1.1 equiv.) in dry DCM was added DMAP (0.11 equiv.) and EDCI (1.1 equiv.). The mixture was stirred at r.t. for 12 h and purified by column chromatography to give the final product (yield = 88%). White solid; ^{1}H NMR (400 MHz, $CDCl_3$) δ 7.83 (dd, *J* = 5.5, 3.1 Hz, 2H), 7.72 (dd, *J* = 5.5, 3.0 Hz, 2H), 6.81 – 6.66 (m, 3H), 4.35 (dd, *J* = 5.7, 4.9 Hz, 2H), 3.96 (dd, *J* = 5.8, 4.9 Hz, 2H), 3.83 (d, *J* = 6.9 Hz, 6H), 3.52 (s, 2H); ^{13}C NMR (101 MHz, $CDCl_3$) δ 171.60, 168.00, 148.80, 148.04, 134.00, 131.92, 126.15, 123.32, 121.46, 112.50, 111.07, 61.78, 55.78, 40.68, 36.96; HRMS (ESI): calcd for: C20H19NO6 $[M+H]^+$ = 370.1291, obsd $[M+H]^+$ = 370.1290.

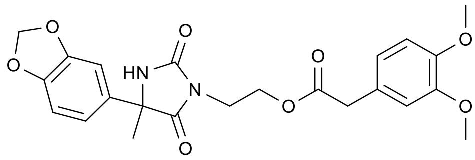

2-(4-(benzo[d][1,3]dioxol-5-yl)-4-methyl-2,5-dioxoimidazolidin-1-yl)ethyl 2-(3,4-dimethoxyphenyl)acetate: Following the method used for **2-(1,3-dioxoisoindolin-2-yl)ethyl 2-(3,4-dimethoxyphenyl)acetate** to give the final product (yield = 41%). White solid; ^{1}H NMR (400 MHz, $CDCl_3$) δ 7.03 – 6.91 (m, 2H), 6.84 – 6.73 (m, 4H), 6.38 (s, 1H), 5.96 (d, *J* = 0.9 Hz, 2H), 4.37 – 4.25 (m, 2H), 3.88 (d, *J* = 11.6 Hz, 6H), 3.84 – 3.76 (m, 2H), 3.48 (s, 2H), 1.74 (s, 3H); ^{13}C NMR (101 MHz, $CDCl_3$) δ 175.17, 171.60, 156.21, 148.88, 148.25, 148.16, 147.81, 132.32, 126.17, 121.60, 118.70, 112.76, 111.20, 108.28, 106.22, 101.46, 63.43, 61.24, 55.92, 40.51, 37.93, 25.12; HRMS (ESI): calcd for: C23H24N2O8 $[M-H]^-$ = 455.1454, obsd $[M-H]^-$ = 455.1452.

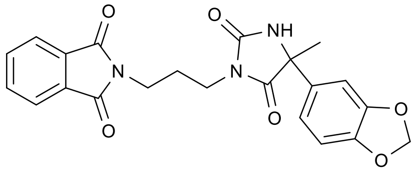

2-(3-(4-(benzo[d][1,3]dioxol-5-yl)-4-methyl-2,5-dioxoimidazolidin-1-yl)propyl)isoindoline-1,3-dione: The mixture of isobenzofuran-1,3-dione (1.0 equiv.) and 3-aminopropan-1-ol (4 equiv.) was refluxed in toluene with NEt_3 for 2 h. Then, the product was stirred with I_2, PPh_3 and imidazole in DCM at r.t. for 4h to give the Iodide. Finally, the mixture of 2-(3-iodopropyl)isoindoline-1,3-dione (1.0 equiv.) and **5-butyl-5-phenylimidazolidine-2,4-dione** (1.0 equiv.) was stirred with K_2CO_3 (2.0 equiv.) at r.t. for 16 h. The reaction mixture was purified by column chromatography to give the final product (yield = 49%). White solid; ^1H NMR (400 MHz, $CDCl_3$) δ 7.88 – 7.76 (m, 2H), 7.75 – 7.65 (m, 2H), 7.05 – 6.93 (m, 3H), 6.83 – 6.73 (m, 1H), 5.92 (d, *J* = 1.0 Hz, 2H), 3.66 (dd, *J* = 17.3, 10.1 Hz, 2H), 3.55 (dt, *J* = 14.0, 7.1 Hz, 2H), 2.04 – 1.98 (m, 2H), 1.79 (s, 3H); ^{13}C NMR (101 MHz, $CDCl_3$) δ 175.39, 168.15, 156.69, 148.17, 147.69, 133.95, 132.45, 132.03, 123.24, 118.75, 108.32, 106.23, 101.37, 63.41, 36.45, 35.36, 27.21, 25.44; HRMS (ESI): calcd for: C22H19N3O6 $[M-H]^-$ = 420.1196, obsd $[M-H]^-$ = 420.1194.

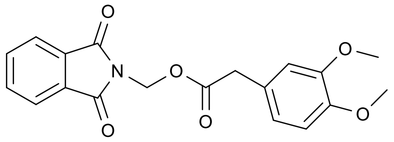

(1,3-dioxoisoindolin-2-yl)methyl 2-(3,4-dimethoxyphenyl)acetate: Following the method used for **2-(1,3-dioxoisoindolin-2-yl)ethyl 2-(3,4-dimethoxyphenyl)acetate** to give the final product (yield = 74%). White solid; ^1H NMR (400 MHz, $CDCl_3$) δ 7.99 – 7.91 (m, 2H), 7.86 – 7.77 (m, 2H), 6.81 (d, *J* = 1.8 Hz, 3H), 5.77 (s, 2H), 3.87 (d, *J* = 7.1 Hz, 6H), 3.60 (s, 2H); ^{13}C NMR (101 MHz, $CDCl_3$) δ 170.60, 166.63, 148.99, 148.29, 134.65, 131.74, 125.64, 123.94, 121.50, 112.39, 111.28, 61.17, 55.89, 40.38; HRMS (ESI): calcd for: C19H17NO6 $[M+H]^+$ = 356.1134, obsd $[M+H]^+$ = 356.1137.

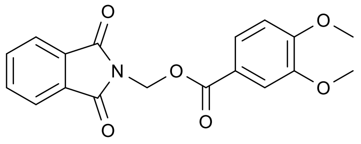

(1,3-dioxoisoindolin-2-yl)methyl 3,4-dimethoxybenzoate: Following the method used for **2-(1,3-dioxoisoindolin-2-yl)ethyl 2-(3,4-dimethoxyphenyl)acetate** to give the final product (yield = 49%). White solid; ^1H NMR (400 MHz, $CDCl_3$) δ 8.02 – 7.94 (m, 2H), 7.82 (dd, *J* = 8.2, 4.2 Hz, 2H), 7.55 (m, 1H), 7.38 – 7.26 (m, 1H), 6.94 – 6.83 (m, 1H), 6.05 – 5.94 (m, 2H), 4.00 – 3.88 (m, 6H); ^{13}C NMR (101 MHz, $CDCl_3$) δ 166.77, 165.16, 153.46, 148.71, 134.61, 131.85, 124.14, 123.97, 121.60, 112.24, 110.26, 61.13, 56.07; HRMS (ESI): calcd for: C18H15NO6 $[M+Na]^+$ = 364.0797, obsd $[M+Na]^+$ = 364.0787.

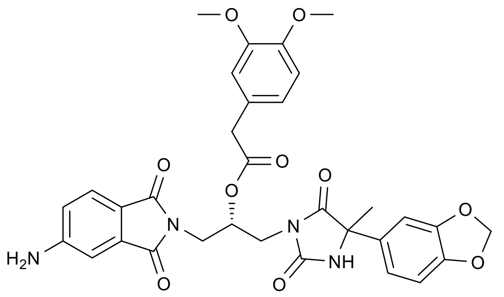

1-(5-amino-1,3-dioxoisoindolin-2-yl)-3-(4-(benzo[d][1,3]dioxol-5-yl)-4-methyl-2,5-dioxoimidazolidin-1-yl)propan-2-yl 2-(3,4-dimethoxyphenyl)acetate: The nitro-substituted **51** was hydrogenated by H_2 and Pd/C. The reaction mixture was purified by column chromatography to give the final product (yield = 71%). Yellow solid; ^{1}H NMR (400 MHz, $CDCl_3$) δ 7.54 (s, 1H), 6.99 – 6.66 (m, 8H), 5.93 (s, 3H), 5.34 (m, 1H), 4.41 (s, 2H), 3.89 – 3.70 (m, 10H), 3.36 (m, 2H), 1.71 (s, 3H); ^{13}C NMR (101 MHz, $CDCl_3$) δ 175.09, 171.31, 168.10, 167.89, 155.87, 152.27, 148.63, 148.23, 147.92, 147.80, 134.53, 132.17, 126.08, 125.19, 121.58, 120.26, 118.81, 117.96, 112.78, 111.00, 108.56, 108.36, 106.25, 101.43, 69.56, 63.35, 55.80, 40.46, 40.12, 38.62, 24.95; HRMS (ESI): calcd for: C32H30N4O10 $[M-H]^-$ = 629.1884, obsd $[M-H]^-$ = 629.1879.
